# The Human SCN10A^G1662S^ Point Mutation Established in Mice Impacts on Mechanical, Heat, and Cool Sensitivity

**DOI:** 10.3389/fphar.2021.780132

**Published:** 2021-12-01

**Authors:** Celeste Chidiac, Yaping Xue, Maria del Mar Muniz Moreno, Ameer Abu Bakr Rasheed, Romain Lorentz, Marie-Christine Birling, Claire Gaveriaux-Ruff, Yann Herault

**Affiliations:** ^1^ CNRS, INSERM Institut de Génétique et de Biologie Moléculaire et Cellulaire (IGBMC), Université de Strasbourg, Illkirch, France; ^2^ CNRS, INSERM, PHENOMIN-Institut Clinique de la Souris, Université de Strasbourg, Illkirch, France

**Keywords:** SCN10A, sodium channel, small fiber neuropathy, nociception, neuropathic pain, statistical modelling

## Abstract

The voltage-gated sodium channel NAV1.8 is expressed in primary nociceptive neurons and is involved in pain transmission. Mutations in the SCN10A gene (encoding NAV1.8 channel) have been identified in patients with idiopathic painful small fiber neuropathy (SFN) including the SCN10A^G1662S^ gain-of-function mutation. However, the role of this mutation in pain sensation remains unknown. We have generated the first mouse model for the G1662S mutation by using homologous recombination in embryonic stem cells. The corresponding Scn10a^G1663S^ mouse line has been analyzed for Scn10a expression, intraepidermal nerve fiber density (IENFD), and nociception using behavioral tests for thermal and mechanical sensitivity. The Scn10a^G1663S^ mutants had a similar Scn10a expression level in dorsal root ganglia (DRG) to their wild-type littermates and showed normal IENFD in hindpaw skin. Mutant mice were more sensitive to touch than wild types in the von Frey test. In addition, sexual dimorphism was observed for several pain tests, pointing to the relevance of performing the phenotypical assessment in both sexes. Female homozygous mutants tended to be more sensitive to cooling stimuli in the acetone test. For heat sensitivity, male homozygous mutants showed shorter latencies to radiant heat in the Hargreaves test while homozygous females had longer latencies in the tail flick test. In addition, mutant males displayed a shorter reaction latency on the 54°C hot plate. Collectively, Scn10a^G1663S^ mutant mice show a moderate but consistent increased sensitivity in behavioral tests of nociception. This altered nociception found in Scn10a^G1663S^ mice demonstrates that the corresponding G1662 mutation of SCN10A found in SFN patients with pain contributes to their pain symptoms.

## Introduction

Neuropathic pain “arises as a direct consequence of a lesion or diseases affecting the somatosensory system” as defined by IASP (Treede et al., 2008; Woolf, 2010; Brouwer et al., 2015). It affects 7% of the general population and 40% of patients with neurological diseases (Bouhassira, 2019). Neuropathic pain is generally characterized by abnormal sensations such as hyperalgesia (increased response to painful stimuli) and allodynia (pain in response to a stimulus that does not normally provoke pain) (Farrar and Portenoy, 2001) and is related to functional changes in primary afferents and sensitization within the central nervous system (CNS) (Brouwer et al., 2015; Bennett et al., 2019). Primary sensory neurons express unique repertoires of voltage-gated sodium channels. These channels are critical in initiating and propagating the all-or-none action potential to the CNS (Bennett et al., 2019). Tissue damage or injury can lead to changes in expression of sodium channel subunits in the sensory neurons, leading to an imbalance between excitatory and inhibitory signaling. These modifications will affect pain thresholds and activity resulting in hyper- or hyposensitivity to pain (Nassar et al., 2005; Colloca et al., 2017).

Inherited disorders of ion channels are known to account for a wide spectrum of human diseases called channelopathies. More than 1,000 disease-related mutations have been identified in the family of NAV channels (NAV1.1-NAV1.9) (Huang et al., 2017). Nowadays, about 800 variants have been described in the SCN10A gene encoding the NAV1.8 channel in the ClinVar NCBI database (https://www.ncbi.nlm.nih.gov/clinvar queried the August 26, 2021), and potentially pathogenic variants of the SCN10A gene were found in 3.7% of patients with small fiber neuropathy (SFN) (Eijkenboom et al., 2019). The missense point mutation c.4984G > A (p.Gly1662Ser) was identified in three SFN patients with years of pain history (Faber et al., 20121944; Han et al., 2014; Eijkenboom et al., 2019). The first patient was 24 years old and suffered from continuous tingling and cramps and reported more pain when exposed to cold temperature or exercising. She had abnormal sensitivity thresholds for warmth and cold stimuli as assessed by quantitative sensory testing (QST). The second patient was 62 years old, experienced continuous burning and stabbing pain, and was very intolerant to sheet. She showed hypoesthesia and reduced vibration sense in physical examination and abnormal warmth sensation levels revealed by QST (Han et al., 2014). The third patient was recently reported. She was 28 years old and showed abnormal temperature sensitivity thresholds (Eijkenboom et al., 2019). In functional studies, the SCN10A^G1662S^ mutation was shown to be the first human disease-causing mutation that enhanced the generation of tetrodotoxin-resistant (TTX-R) resurgent currents (Xiao et al., 2019), impaired channel inactivation, and accelerated recovery from inactivation, which result in hyperexcitability of DRG neurons (Han et al., 2014).

With the aim to study the causality of the SNC10A G1662S mutation in pain, we have generated the corresponding mouse model. The comparison of human and mouse SCN10A protein sequences showed 82% homology with a resultant of one additional amino acid found in the mouse vs. human sequence before the G1662 residue, leading to a change in the position of the human G1662 to G1663 in mice (see Supplementary Figure S1). We have created the Scn10a^G1663S^ mouse model corresponding to the G1662S mutation in the human gene and have investigated the p.G1662S genotype–phenotype link and in order to relate this mutation to SFN pain symptoms. The Scn10a transcripts are well expressed in the DRG of mutant mice as compared to wild-type controls. The mutant animals showed no alteration of growth, survival, and health state. The Scn10a^G1663S^ mutant mice of both sexes were evaluated for pain behavioral responses to noxious cold, heat, and mechanical stimuli. We found that Scn10a^G1663S^ mutant mice displayed a moderate pain phenotype and identified the behaviors contributing the most to the pain phenotype using a novel statistical modeling analysis. This suggests that the SCN10A^G1662S^ mutation identified in SFN patients with pain favors their pain symptoms.

## Materials and Methods

### Animals

#### Experimental Subjects and Ethical Approval

Animal experiments were performed after approval from the local ethical committee (Com’Eth, “Comité d’Ethique pour l’Expérimentation Animale IGBMC-ICS”) with the agreement number 14691. The number of mice used for the experiments was adapted according to the 3R principles. Mouse breeding and behavioral experiments were conducted in SPF conditions at the ICS animal facility. Animals were housed under a 12-h/12-h light/dark cycle (lights on at 7 a.m.) and 21 ± 1°C, 55 ± 10% humidity condition. Standard Chow (D04 for maintenance and D03 for breeding, SAFE, Les Oncins, France) and autoclaved tap water were provided ad libitum with two to five mice housed per cage. Prior to beginning behavioral experiments, all mice were habituated to the environmental conditions and to handling.

### Establishment of the Genetic Animal Model

The mouse line creation was done on the C57BL/6NCrl genetic background. Mutant mice carrying the G1663S mutation in the Scn10a gene corresponding to the G1662S mutation in the human gene (Supplementary Figure S1) were created by homologous recombination in ES cells. A targeted allele was engineered bearing two homology arms of the original Scn10a endogenous gene and a floxed Neo resistance auto-selection cassette. A 1.2-kb fragment encompassing Scn10a exon 28 (ENSMUSE00000582971) and 5′ intronic sequence was amplified by PCR on ES cell C57BL/6N genomic DNA in two steps to allow the introduction of the GGC > AGC point mutation (leading to the G1663S mutation) and subcloned in an ICS proprietary vector. The 5′ arm of homology (1.1 kb) which consisted of part of intron 27 was amplified by PCR and subcloned in a step1 plasmid to generate the final targeting construct. An RNA guide, gR68 (guide RNA sequence CTA​ATA​AGA​TAT​TCT​GGG​TA), targeting the intronic sequence at the site of insertion of the NeoR selection cassette was cloned in pX330 from Addgene (CRISPR/Cas9 plasmid). This vector was used to increase the efficiency of homologous recombination at the Scn10a locus.

**FIGURE 1:**
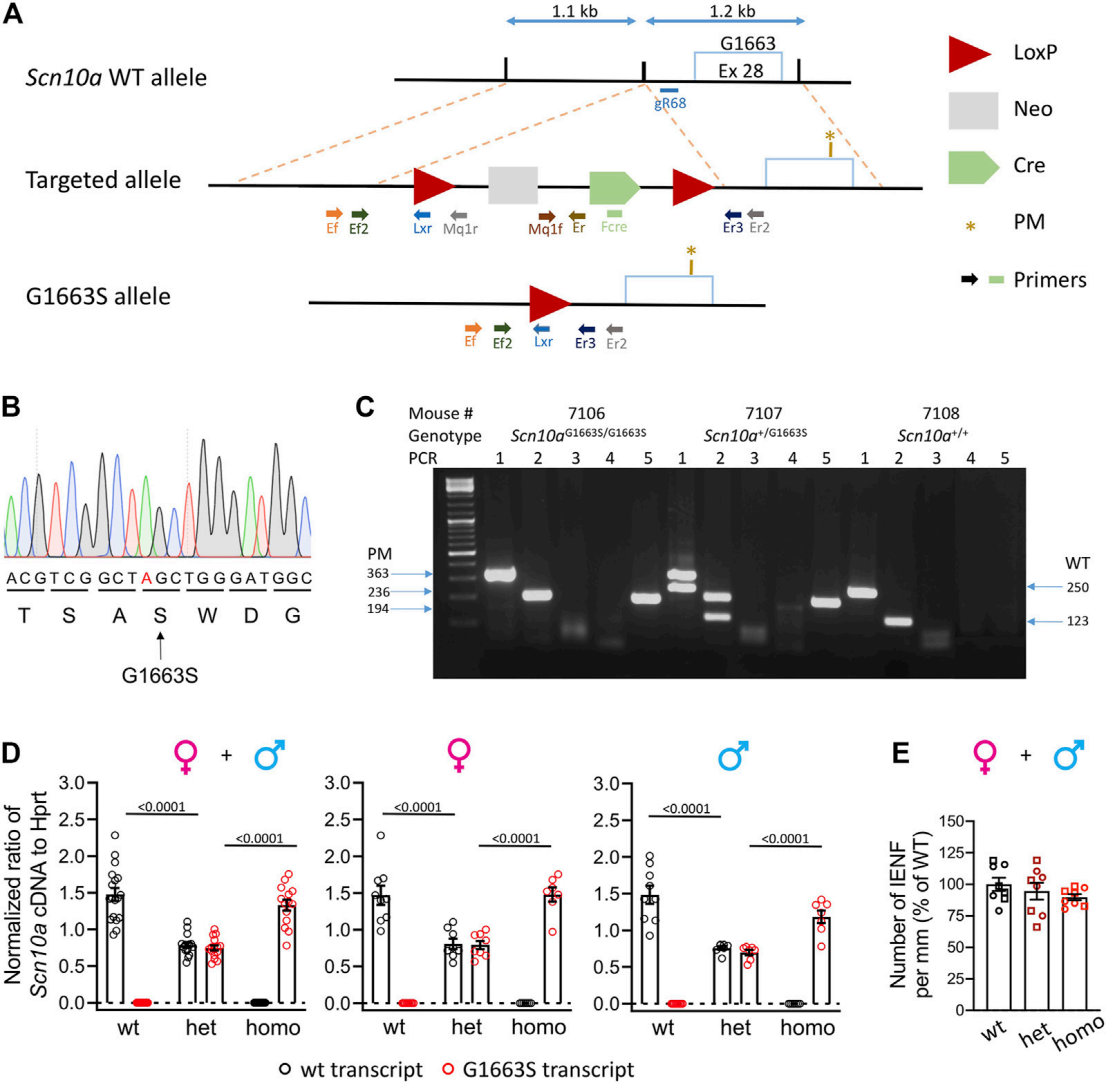
Generation and validation of the Scn10a^G1663S^ mouse model. **(A)** Targeting strategy by homologous recombination. The scheme shows the Scn10a WT allele, the targeted allele, and the G1663S knock-in allele. The targeted allele was engineered bearing two homology arms of the original Scn10a endogenous gene and an auto-selection cassette. The 3′ arm of homology (1.2 kb) consisted of a part of intron 28 and of a part of exon 28 bearing the S1663 mutation. The 3′ arm was cloned into a targeting vector containing the loxP-flanked (“floxed”) neo cassette. The 5′ arm of homology (1.1 kb) consisted of part of intron 28 and was cloned into the vector containing the auto-selection cassette and the 3′ arm. The final mutant allele was obtained after excision of the selection cassette. Light blue box: Exon 28 containing the locus G1663/S1663; the PM was indicated with a brown star; blue double arrow lines: indicate size of the arms; red triangles: LoxP sites; light gray box: neomycin selection cassette; green pentagon: Cre cassette. Different-colored lightning block arrows show the primers used for genotyping. The green rectangle indicates the primer located in the Cre cassette (Fcre) used for DNA sequencing. **(B)** Mutant allele sequence characterized through Sanger sequencing on a positive ES clone using a specific primer located in the Cre cassette (Fcre) amplifying only the mutant allele. The mutated nucleotide is indicated in red. **(C)** Example of genotyping results on electrophoresis gel showing three different genotypes. For all samples, PCR3 and 4 gave no bands indicating that the selection marker was excised. For sample #7106: PCR1 and 2 gave one band each at 363 and 236 bp, respectively. PCR5 gave one band of 194 bp. These reactions confirm that one LoxP site is still there and the Scn10a^G1663S/G1663S^ genotype. For sample #7107: PCR1 and 2 gave two bands each at 363 and 250 bp and 236 and 123 bp, respectively. One band at 194 bp for PCR5. These PCR reactions confirm that one LoxP site is still there and the Scn10a^+/G1663S^ genotype. For sample #7108: PCR1 and 2 gave one band each at 250 and 123 bp, respectively. No band for PCR5, confirming the wild-type genotype. **(D)** Wild-type and G1663S allele mRNA expression in DRG of Scn10a^G1663S^ heterozygous and homozygous mutant mice. Scn10a mRNA expression was normalized to Hprt expression. Homozygous mutant mice expressed Scn10a^G1663S^ transcript at levels comparable to wt transcript expression, and heterozygous mice express half level of each transcript type. Females, *n* = 7–9/group; males, *n* = 7–9/group. One-way ANOVA analysis was followed by Sidak’s multiple-comparison tests. The bars and *p* values indicate significant differences (*p* < 0.05) between wt and heterozygous mutant mice, and between heterozygous and homozygous mutant mice in Sidak’s multiple-comparison tests. See Supplementary Table S3 for detailed statistical analysis. **(E)** The G1663S mutant mice showed comparable IENFD compared to wt. Data are expressed as % of the mean of IENFD of each animal to the mean of wt of the same sex. Circles are used for females and squares for males. *n* = 4 mice per genotype and sex. The two-way ANOVA for the effects of genotype and sex as well as one-way ANOVA on grouped sexes showed no sex or genotype effect. Data are expressed as means ± SEM in all panels. See Supplementary Table S4 for detailed statistical analysis.

The homologous recombination strategy is shown in Figure 1A. Both plasmids targeting plasmid and CRISPR/Cas9 plasmid were electroporated in C57BL/6N mouse ES cells (ICS proprietary line). After G418 selection, targeted clones were identified by long-range PCR and further confirmed by Southern blot with an internal (Neo) probe and a 5′ external probe.

We confirmed Neo cassette insertion by long-range PCR and verified that the construct was not integrated randomly in the genome. Clones with the correct genotype were injected into BALB/CN blastocysts, and resulting chimeric males having the correct targeted genotype and a color score >85% were bred with C57BL/6NCrl WT females for germline transmission. Heterozygous Scn10a^+/G1663S^ male and female mice were crossed to generate the heterozygous Scn10^+/+^ (= wt), heterozygous Scn10^+/G1663S^ (het), and homozygous Scn10a^G1663S/G1663S^ (homo) mice.

### Determination of Genotype

For genotype determination, 0.5 cm of mouse tails was harvested. Genomic DNA was extracted by DirectPCR Lysis Reagent Tail (Viagen Biotech, Los Angeles, CA, USA; Cat # 101-T) according to the manufacturer’s instructions. Five hundred nanograms of genomic DNA extracted from WT or mutant mouse was PCR-amplified by 0.2 µl Taq DNA polymerase (Roche), 1.5 µl 10× PCR buffer with MgCl_2_, 0.3 µl dNTP PCR-grade nucleotide (dATP, dCTP, dGTP, dTTP at 10 mM, Thermo Scientific, Waltham, MA, USA), 0.5 µl each primer at 10 µM, and H_2_O in a total volume of 20 ml. Using a T100 thermocycler (Bio-Rad, Hercules, CA, USA), PCRs were subject to the following thermal conditions: a denaturation step at 94°C for 2 min followed by 40 cycles of 94°C for 30 s, a gradient of annealing temperatures between 55°C and 65°C for 45 s and 72°C for 1 min/kb, and a final elongation step for 5 min at 72°C. The PCR outcome was analyzed on a 1.5% agarose gel. To confirm the G1663S mutation, genomic DNA from mutated mice was PCR-amplified and confirmed by DNA sequencing. Genotyping of G1663S mutant mice consisted of five PCR reactions: PCR1 and PCR2 to confirm the excision of the selection marker using Ef/Er2 and Ef2/Er3 primers and giving bands of 4,484 and 4,357 bp for the recombinant allele, 363 and 236 bp for the PM allele, and 250 and 123 bp for the WT allele, respectively. PCR3 and PCR4 targeting the 5′ and 3′ sides of the selection marker, respectively, using Ef/Mq1r and Mq1f/Er primers giving respectively bands of 286 and 461 bp for the targeted allele and no bands for PM and WT alleles. The PCR5 specific for the LoxP site using Ef/Lxr primers gave a band of 194 bp for the targeted and PM alleles. Primer sequences and PCR reaction information are shown in Supplementary Tables S1A,B, and primers cutting sites are shown in the homologous recombination strategy in Figure 1A.

### Determination of Transcript Expression by Real-Time Droplet Digital PCR

Dorsal root ganglia were collected from WT and mutant mice and flash frozen in liquid nitrogen. All tissues were disrupted using Precellys^®^ CK14 Lysing Kit in TRIzol Reagent and total RNA purified using RNeasy Mini Kit (Qiagen, Hilden, Germany) according to the manufacturer’s instructions. cDNA synthesis was performed using the SuperScript™ VILO™ cDNA Synthesis Kit (Invitrogen, Carlsbad, CA, USA). The Scn10a transcription level was determined by using ddPCR and Hprt as a standard gene. ddPCR was performed in 20-µl reactions containing 1× ddPCR Supermix for Probes (No dUTP), 125 nM of each probe, 250 nM specific primers, and 50 ng DNA according to the manufacturer’s recommendations (PCR conditions: 10 min at 95°C, 40 cycles of 20 s at 95°C 30 s at 59.2°C, 2 min at 72°C; and 10 min at 98°C). Droplet generation and absolute droplet quantification were performed in a QX200 Droplet Digital PCR System (Bio-Rad) and analyzed by QuantaSoft Software (Bio-Rad). Probe and primer sequences are shown in Supplementary Table S2.

### Histological Analysis

#### Tissue Preparation

Naïve adult mice (aged 23–28 weeks) were used for the histological analysis. Mice were anesthetized with ketamine/xylazine and perfuse intracardially with 40 ml of phosphate-buffered saline 0.1 M pH 7.4 (PBS) following 4% paraformaldehyde (PFA) in 0.1 M pH 7.4 PBS. Glabrous hindpaw skin samples (5 mm long) were carefully dissected out and postfixed for overnight at 4°C in 4% PFA in PBS, then tissues are removed from PFA and cryoprotected at 4°C in 30% sucrose in phosphate buffer (PB) for 3 days, embedded in optimal cutting temperature medium, frozen, and kept at −80°C. Glabrous skin longitudinal sections (30 µm thick) were cut with a cryostat and kept at −20°C.

### Immunohistochemistry

IHC of skin sections was performed with free-floating sections. Light antigen retrieval was performed in 0.1 M PB pH 7.3, 0.2% hydrogen peroxide, and 0.05% Triton X-100 for 25 min at room temperature (RT). Sections were then washed two times in PBS and incubated in blocking solution of Tris-buffered saline (TBS) with 0.05% Tween 20, 2% bovine serum albumin (BSA), and 2% normal donkey serum (NDS) for 1 h before applying a primary antibody overnight at 4°C. The following primary antibody (diluted in the blocking solution) was used: rabbit anti-PGP9.5 (1:500, ab108986, Abcam). For detection of the primary antibody, a secondary antibody raised in donkey and conjugated with Alexa-647 fluorophore was used (1:500, Molecular Probes, Thermo Fisher Scientific) for 1 h at RT. DAPI staining (1:2,000, Molecular Probes) was performed as the same time as the secondary antibody. Sections were then washed two times with PBS then placed on superfrost glass slides. One drop of Immu-Mount is added over the tissue sections, and a cover glass is placed over the slide.

### Image Acquisition and Analysis

N = 4 mice per genotype and sex were used for IENFD determination. Both hindpaw skin and four randomly selected sections per paw were analyzed. Images were visualized with Leica SP8 UV/Visible Laser Confocal Microscope using a ×20 oil objective. The ×20 resolution was achieved with a digital zoom factor of 3. Image acquisitions in the sequential mode (excitation intervals: 415–550 nm and 643–750 nm) were used for marker co-localization to avoid potential cross talk between the different fluorescence emissions. Images were acquired with the LCS (Leica, Wetzlar, Germany) software. The whole length of the skin sections was acquired as a tile scan, and an overview of the epidermis–dermis was reconstructed by acquisition of z-stacks with a total depth of 30 μm. IENF were counted, and the length of the epidermal surface is measured using ImageJ software on digitized confocal images (Schneider et al., 2012). Counting on blinded samples was performed manually on five randomly chosen sections per paw per animal. Afferents crossing the dermal–epidermal junction were counted. Nerves that branch after crossing the junction were counted as a single unit. Nerves that split below the junction or appear to branch within the junction were counted as two units (Lauria et al., 2005). IENFD was determined by dividing the number of IENF by the total length of skin analyzed per section. We defined for each animal the IENFD as the mean of the IENFD calculated for the four sections per animal. The % of IENFD in mutants was calculated by dividing the mean of IENFD of each mouse by the mean IENFD of wt mice of the same sex.

### Behavioral Assessment of the Scn10a^G1663S^ Mouse Model

A pipeline for behavioral assessment was defined and conducted on mutants and control littermates to investigate nociception and proprioception. All behavioral tests were conducted between 9:00 a.m. and 5:00 p.m. Animals were transferred to the experimental room 30 min before each experimental test. Body weights of wt and mutant mice were measured at the age of 7–11 weeks. The behavioral tests were carried on from the least noxious to the most noxious test in the following order: string test, crenellated bar, von Frey, Hargreaves plantar, acetone test, tail flick, tail pressure, cold plate, and hot plate. At least 2 days were kept between two consecutive tests. Females and males of the different genotypes were tested at 2 months of age. The experimenter was blind to mouse genotype.

The grip string test (in-house) was used to measure muscle strength. It was built as a horizontal wire placed at 40 cm above a table. The mouse was hanged by the forelimb to the wire, and the latency to hindlimb traction was measured. Three consecutive trials were done by 5-min intervals.

The notched/crenellated bar test was used to evaluate motor coordination and balance. The method described by Carter et al. was used (Carter et al., 2001). Briefly, mice were put on an elevated crenellated bar and had to traverse the distance from the beginning of the bar until the end where the home cage is placed. The time required to traverse the whole bar distance was recorded. In addition, the number of mistakes was defined as the times when mice were touching the gaps of the crenelated bar by their hindpaws while passing over the bar.

Tactile sensitivity was analyzed with the Von Frey test. One day prior to the test, mice were placed in small transparent plastic boxes over a mesh floor and were left there to acclimatize for 45 min. On the test day, mice were habituated to the box for 30 min before starting the test. Mechanical sensitivity was determined using different von Frey filaments of specific forces from 0.008 to 2 g (Bioseb, Vitrolles, France) and the “up–down” von Frey method (Weibel et al., 2013). Briefly, filaments were applied to the mid-plantar hindpaws starting with the filament of 0.4 g. Each trial was done by applying the filaments onto one hindpaw of mice placed consecutively and then applying the filaments onto the other hindpaw of each mouse. The 50% mechanical threshold was calculated for each animal. The test was reproduced on two consecutive days with two trials per day.

The “Rodent pincher” test (Bioseb, France) was used to determine sensitivity to noxious mechanical stimuli, by applying a gradually increasing pressure to mice tail. This test allows calibrated forceps to induce quantifiable mechanical stimulation on a linear scale with a cutoff of 500 g. Two days prior to the Rodent pincher test, animals were habituated to be restrained in a 50-ml tube, with the whole tail exposed. Three measures were performed for each mouse, recording on three different locations of the tail. The mean of tail withdrawal threshold was calculated.

The acetone test was used to measure sensitivity to cool stimuli. Mice were placed and habituated in the same manner as for the von Frey test. Ten microliters of acetone was applied to the center of the plantar surface of each hindpaw, using a 50 µl Hamilton Gastight Syringe (Model 1705 TLL). Acetone was applied in three successive testing sessions for each paw. The interval between each application was at least 5 min. The duration of hindpaw withdrawal and flicking was measured during 30 s following acetone application. The values of hindpaw withdrawal and flicking duration displayed in the six trials were added.

The cold plate assay (Bioseb, France) was used to assess sensitivity to noxious cold temperatures. One day prior to the test, mice were acclimatized to the plate at RT, then on the test day, mice were tested on a 5°C cold plate. To avoid tissue damage, a maximum testing time of 5 min was set up. The mean numbers of hindpaw lifts and jumps were measured.

The Hargreaves test consisted of applying a radiant infrared heat stimulus to the hindpaws of mice to determine heat thresholds. One day prior to the test, mice were habituated to the apparatus by placing them in small transparent plastic boxes over a glass plate. Withdrawal latency was recorded on two consecutive days, with two trials per day. A trial was done by applying the heat stimulus on one hindpaw for all the mice, then applying it to the second hindpaw for all the mice, leaving at least 5 min between each hindpaw recording of the same mice. The mean of all measurements of both wt paws was calculated. The radiant heat was applied using a temperature ramp speed allowing to get a paw withdrawal latency of 7 s in wt mice.

Tail flick consisted in applying a focused beam of light to the mouse tail, to determine heat thresholds. Two days prior to the test, mice are habituated to be restrained in a 50-ml tube, with the whole tail exposed. The heat stimuli were applied at three different places of the tail tip, and the mean of the three withdrawal latencies was calculated.

A hot plate test was used as an integrated test to determine heat sensitivity. One day before the test, mice were habituated to the plate at RT. In our study, the mice were tested at three different temperatures on 3 separate days with specific cutoffs to avoid tissue damage: 3 min at 47°C, 1 min at 50°C, and 30s 54°C. At each temperature, the latency to display first a reaction was recorded. In addition, we also measured coping reactions, a second line behavioral response to pain in addition to reflex responses, as reported recently by Huang et al. (Huang et al., 2019). Mutant mice with ablated spinal preprotachykinin-positive neurons showed withdrawal latencies comparable to their wt littermates on the hot plate while they showed reduced number of licking episodes compared to wts (Huang et al., 2019), revealing the importance of scoring coping behaviors as an additional information of heat pain sensitivity in this test. In our study, the coping behaviors included lifting, flicking, licking of the hindpaws, and jumping. The number of total reactions was calculated per minute of test at each temperature.

### Statistical Analysis

Results are expressed as mean ± SEM. Statistical analyses were performed by using GraphPad Prism 9 software. All data sets were tested for normality using the D’Agostino and Pearson normality test. One-way ANOVA analysis was done when data were found normally distributed, and Kruskal–Wallis analysis was used when data had non-normal distribution. For transcript expression level analysis, the genotype effect in each sex was analyzed using one-way ANOVA analysis followed by Šídák’s multiple-comparison test when appropriate. The analysis of behavioral tests results was performed by one-way ANOVA or Kruskal–Wallis analyses to test for genotype difference on grouped sexes and separated sexes, followed by *post-hoc* analysis when appropriate. A *p*-value < 0.05 was considered statistically significant. Supplementary statistical analyses were done to identify the variables contributing the most to the genotype or sex discrimination using the Gdaphen R pipeline. The principle of Gdaphen analysis is described in the Supplementary Material.

## Results

### Generation and Characterization of the Scn10a^G1663S^ Mouse Model

Initially, we tried to create the Scn10a^G1663S^ mouse model with the CRISPR-Cas9 innovative technology. We tried two different strategies that were unsuccessful due to the high similarity between Scn10a and other Scn genes, especially the Scn5a gene that shares the highest similarity with Scn10a and is expressed on the same chromosome. We moved then to homologous recombination in ES cells to generate the Scn10a^G1663S^ mouse line (Figure 1A). Briefly, the G1663S-targeting vector was electroporated into C57BL/6N ES cells, together with the plasmid vector containing a guide RNA named gR68. The guide RNA gR68 in association with the Cas9 protein specifically generates a double-strand break in the intronic region close to the targeted region. The intronic sequence, in contrast to the exonic sequence, does not show any similarity to intronic sequences of any other Scn genes. The CRISPR/Cas9 double-strand break driven by the guide RNA gR68 forced the ES cells to repair, and the presence of the targeting construct with the selection pressure (NeoR) increased the homologous recombination rate (even if the size of the homology arms is much less important than with classical ES cell targeting). Among positive ES clones, one clone was selected. The genomic DNA from the mutant allele of this clone was PCR-amplified with a forward primer located in the Cre cassette (Fcre) (Figure 1A) and a 3′ external reverse primer and was then sequenced. Sequencing results demonstrated the presence of the mutation in this positive ES clone (Figure 1B). Heterozygous Scn10a^+/G1663S^ mice were crossed to generate the Scn10a^G1663S/G1663S^ mice and their control wt mice. An example of genotyping results is shown in Figure 1C. The Scn10a transcript expression level was evaluated in the DRG of G1663S and control mice by RT-ddPCR. One-way ANOVA revealed a genotype effect for each allele-derived (wt or mutant G1663S) transcript expression in grouped or separate sexes. When ratios of Scn10a^+^ and Scn10a^G1663S^ transcripts were summed, no effect on genotype or sex was detected by the two-way ANOVA. Both transcripts were equally expressed in the DRG of wt or G1663S/G1663S mutant mice respectively and in both sexes, indicating that the mutation did not alter the Scn10a expression. Heterozygous mice expressed about 50% of each allelic transcript in DRG (Figure 1D and Supplementary Table S3). SFN patients with heterozygous c.4984G > A, p.G1662S mutation showed a normal IENFD. We have quantified IENFD in adult control and mutant mice. No genotype or sex effect was detected in the two-way ANOVA test. The one-way ANOVA on grouped sexes also showed no difference between mutant and wt animals (Figure 1E and Supplementary Table S4).

### Normal Body Weight and Proprioception in the Scn10a^G1663S^ Mice

**FIGURE 2:**
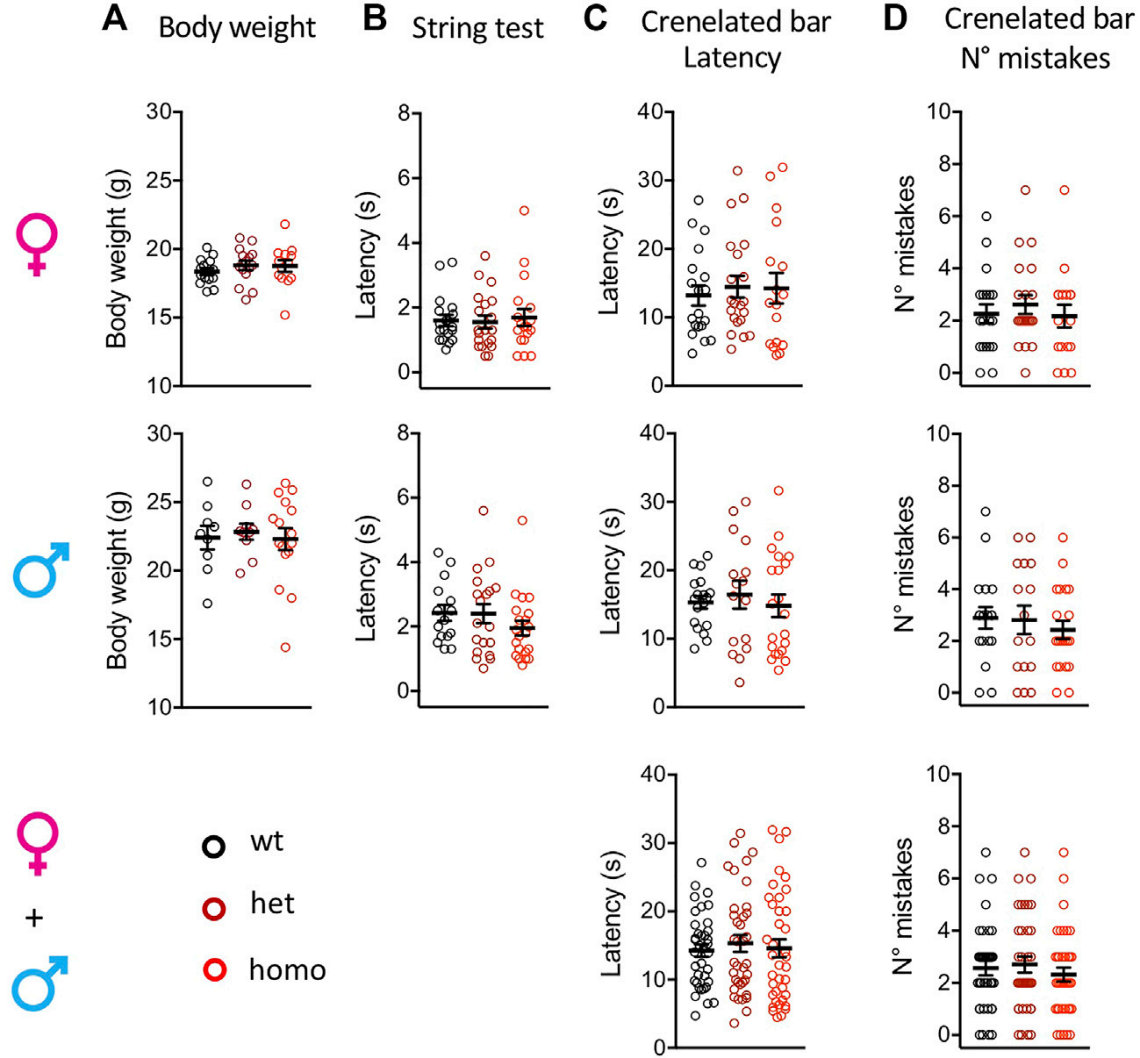
Scn10a^G1663S^ mice show normal healthy conditions and proprioception capacities. **(A)** Body weight of Scn10a^G1663S^ mice measured at the age of 7–11 weeks. Females, *n* = 13–15/group; males, *n* = 9–16/group. The one-way ANOVA for genotype on separated sexes showed no genotype effect. **(B)** Muscle strength was evaluated by string test. Females, *n* = 18–20/group; males, *n* = 15–21/group. A sex effect was detected in the string test. The Kruskal–Wallis test on separated sexes showed no genotype effect. **(C-D)** Motor coordination and balance were evaluated by the latency and the number of mistakes in the crenellated bar test. Females, *n* = 17–21/group; males, *n* = 16–21/group. The Kruskal–Wallis tests on separated or grouped sexes showed no sex or genotype effect. Data are expressed as means ± SEM in all panels. See Supplementary Table S5 for detailed statistical analysis.

The Scn10a^G1663S^ mutation did not affect the body weight of the mutant mice. Heterozygous +/G1663S and homozygous G1663S/G1663S mice showed a normal body weight compared to wt littermates (Figure 2A and Supplementary Table S5). To evaluate whether the mutation alters the proprioception of mutant mice, string test and crenelated bar were performed. String test latency analysis revealed a sex effect (two-way ANOVA F_1, 105_ = 10.76, *p* = 0.0014) but no genotype effect. Similarly, no genotype or sex effect was detected in the crenelated bar test. Heterozygous and homozygous mutant mice behaved in the same manner than control mice in these two tests (Figure 2B-D and Supplementary Table S5). We concluded that the presence of one or two alleles with the G1663S mutation did not change body weight and proprioception in the mutant mice.

### Enhanced Sensitivity to Mechanical Stimuli in the Scn10a^G1663S^ Mice

**FIGURE 3:**
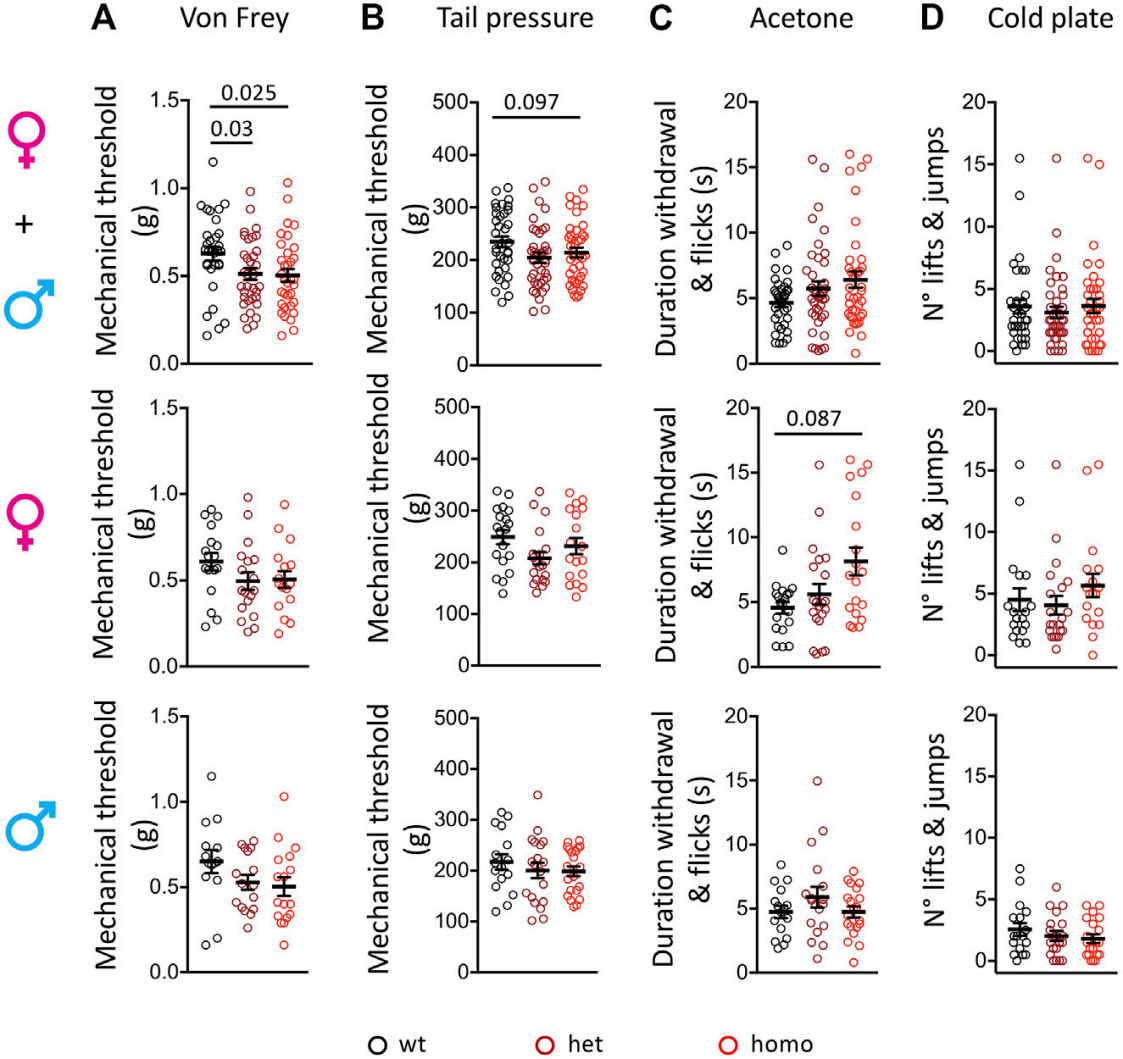
Scn10a^G1663S^ mice show enhanced pain sensitivity to mechanical and cool stimuli. **(A)** G1663S mutant mice displayed hypersensitivity to von Frey filaments. Females, *n* = 17–18/group; males *n* = 14–17/group. The one-way ANOVA showed a general genotype effect in sex-grouped animals. Unpaired two-tailed t-tests showed a genotype effect for wt vs. heterozygous and wt vs. homozygous mutant sex-grouped mice. The 2 bars and *p* values are for genotype difference in grouped females and males, wt vs. heterozygous and wt vs. homozygous mutants. **(B)** Noxious mechanical sensitivity was analyzed by the tail pressure test. Females *n* = 18–20/group; males *n* = 16–20/group. Kruskal–Wallis analysis indicated a trend for genotype effect in sex-grouped animals. The bar and *p* value represent the overall genotype effect in grouped females and males. **(C)** Sensitivity to cool temperature was analyzed by the acetone test. Females *n* = 19–21/group; males *n* = 16–20/group. Mutant females tended to display longer duration of paw reactions following acetone application. Kruskal–Wallis analysis. The bar and *p* value represent the overall genotype effect in females. **(D)** G1663S mutant mice showed no phenotype for cold response on the 5°C cold plate. Females *n* = 18–21/group; males *n* = 17–20/group. Kruskal–Wallis analysis showed no genotype effect in either sex-grouped or sex separated animals. Data are expressed as means ± SEM in all panels. See Supplementary Table S6 for detailed statistical analysis.

The oldest SFN patient with heterozygous G1662S mutation developed sensitive skin and intolerance to sheets over her feet with continuous pain (Han et al., 2014; Eijkenboom et al., 2019). We used the von Frey test to assess touch allodynia and the rodent pincher test to evaluate sensitivity to noxious mechanical stimuli in the Scn10a^G1663S^ mutant mice. In the von Frey test, a genotype effect was present (two-way ANOVA F_2, 93_ = 3.461, *p* = 0.035). The one-way ANOVA showed a genotype effect on grouped sexes where heterozygote and homozygote mice showed a higher sensitivity to mechanical stimuli compared to their wt littermates (Figure 3A and Supplementary Table S6). This did not reach significance when males and females were analyzed separately. In the rodent pincher test, two-way ANOVA analysis resulted in a sex effect (two-way ANOVA F_1, 106_ = 4.186, *p* = 0.043). In addition, there was a tendency for higher sensitivity in the mutants that was above the significance threshold (Figure 3B and Supplementary Table S6). Therefore, Scn10a^G1663S^ mutant mice developed mechanical allodynia.

### Sensitivity to Cooling and Cold Stimuli in the Scn10a^G1663S^ Mice

One of the SFN patients with G1662S mutation reported that cold temperature aggravated her complaints and QST revealed abnormal thresholds for cold sensation (Han et al., 2014). We performed the acetone test to investigate the sensitivity of the Scn10a^G1663S^ mice to cooling stimuli (12°C–15°C) and cold plate at 5°C for cold stimuli. In the acetone test, the duration of withdrawal and flicking reactions revealed an interaction between genotype and sex (two-way ANOVA F_2, 107_ = 4.336, *p* = 0.015). The Kruskal–Wallis analysis showed a higher behavioral response to the acetone stimulus in mutant females while mutant males reacted comparably to wt males (Figure 3C and Supplementary Table S6). In the cold plate test, the number of hindpaw lifts and jumps showed a sex effect (two-way ANOVA F_1, 107_ = 21.61, *p* < 0.0001) with fewer reactions in males. Mutant and control mice of both sexes had comparable reactions on the cold plate (Figure 3D and Supplementary Table S6). Therefore, Scn10a^G1663S^ mutant mice showed a higher sensitivity to cool, but not cold temperatures.

### Enhanced Heat Pain Sensitivity in the Scn10a^G1663S^ Mice

**FIGURE 4:**
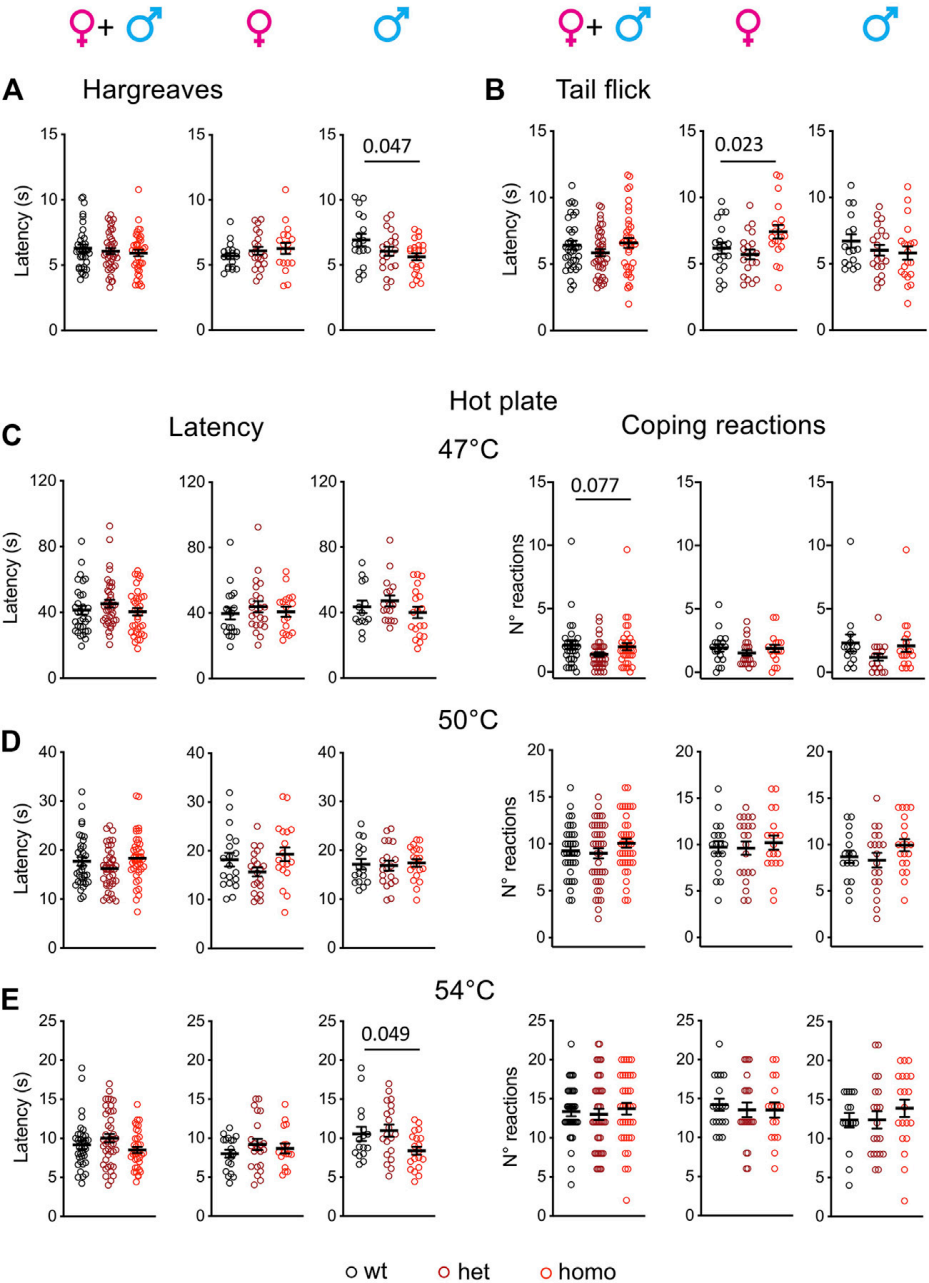
Scn10a^G1663S^ male mice show enhanced pain sensitivity to heat stimuli. **(A)** Male mutants show increased heat sensitivity in the Hargreaves test. Females *n* = 18–21/group; males *n* = 18–22/group. One-way ANOVA indicated a genotype difference in males and Sidak’s multiple-comparison test showed a significant difference in wt vs. homozygous males. The bar and *p* value represent the genotype effect in wt vs. homozygous males. **(B)** Mutant females had increased latency in the tail flick test. Females *n* = 19–20/group; males *n* = 16–20/group. The one-way ANOVA showed an overall genotype difference in females. The bar and *p* value represent the global genotype effect in females. No genotype effect could be seen in wt vs. heterozygous or wt vs. heterozygous females. **(C-E)** Hot plate test. The latency to the first hindpaw reaction is shown in left panels and the number of coping reactions in right panels. **(C)** Mutant mice showed no phenotype on the 47°C hot plate. *n* = 17–22/group; males *n* = 14–19/group. Kruskal–Wallis analysis on sex-separated or grouped animals showed no genotype difference for first hindpaw sign latency and a trend for coping reactions. The bar and *p* value represent the global genotype effect for coping reaction in sex-grouped animals. **(D)** Mutant males or females showed no genotype difference on the 50°C hot plate. Females *n* = 19–21/group; males *n* = 16–20/group. One-way ANOVA on sex-separated or grouped animals. **(E)** The male mutants were more sensitive than their wt counterparts on the 54°C hot plate. Females *n* = 17–22/group; males *n* = 16–20/group. Kruskal–Wallis analysis showed a global genotype effect in males. The bar and *p* value represent the global genotype effect in males. Data are expressed as means ± SEM in all panels. See Supplementary Table S7 for detailed statistical analysis.

An abnormal threshold for warmth sensation was also an important clinical feature of SFN patients with the G1662S mutation (Han et al., 2014; Eijkenboom et al., 2019). Scn10a^G1663S^ mutant mice were tested for their sensitivity to radiant heat stimuli in the Hargreaves and tail flick tests (Figure 4). The statistical analysis of the latencies in the Hargreaves and tail flick tests identified an interaction between sex and genotype (two-way ANOVA F_2, 113_ = 3.808, *p* = 0.025 and F_2, 108_ = 3.345, *p* = 0.039 respectively). In the Hargreaves test, the mutation lowered response latency, with homozygous males withdrawing their hindpaw earlier than wt males (Figure 4A and Supplementary Table S7). In the Tail Flick assay, female mutant mice showed a general genotype effect (Figure 4B and Supplementary Table S7).

The hot plate test is classically used to determine heat sensitivity. Latencies to first hindpaw reaction and to first jump reaction are important variables in this assay. Huang and others introduced the scoring of the number and duration of licking and jumping as outcomes of coping behaviors (Huang et al., 2019). Accordingly, we scored both the latency to first hindpaw reaction at 47°C, 50°C, and 54°C and the number of coping reactions (Figures 4C–E). The latency to first hindpaw reaction on the hot plate showed a sex effect at 54°C (two-way ANOVA F_1, 108_ = 5.703, *p* = 0.019), and a genotype effect was detected at 54°C for the homozygous males (Figure 4E and Supplementary Table S7). For the coping reactions, a tendency for genotype effect was recorded at 47°C although it did not reach significance (Figure 4C and Supplementary Table S7). Therefore, Scn10a^G1663S^ mutant male mice showed an increased sensitivity to heat.

### Gdaphen Analysis for the Identification of the Variables Contributing the Most to the Genotype or Sex Discrimination

**FIGURE 5:**
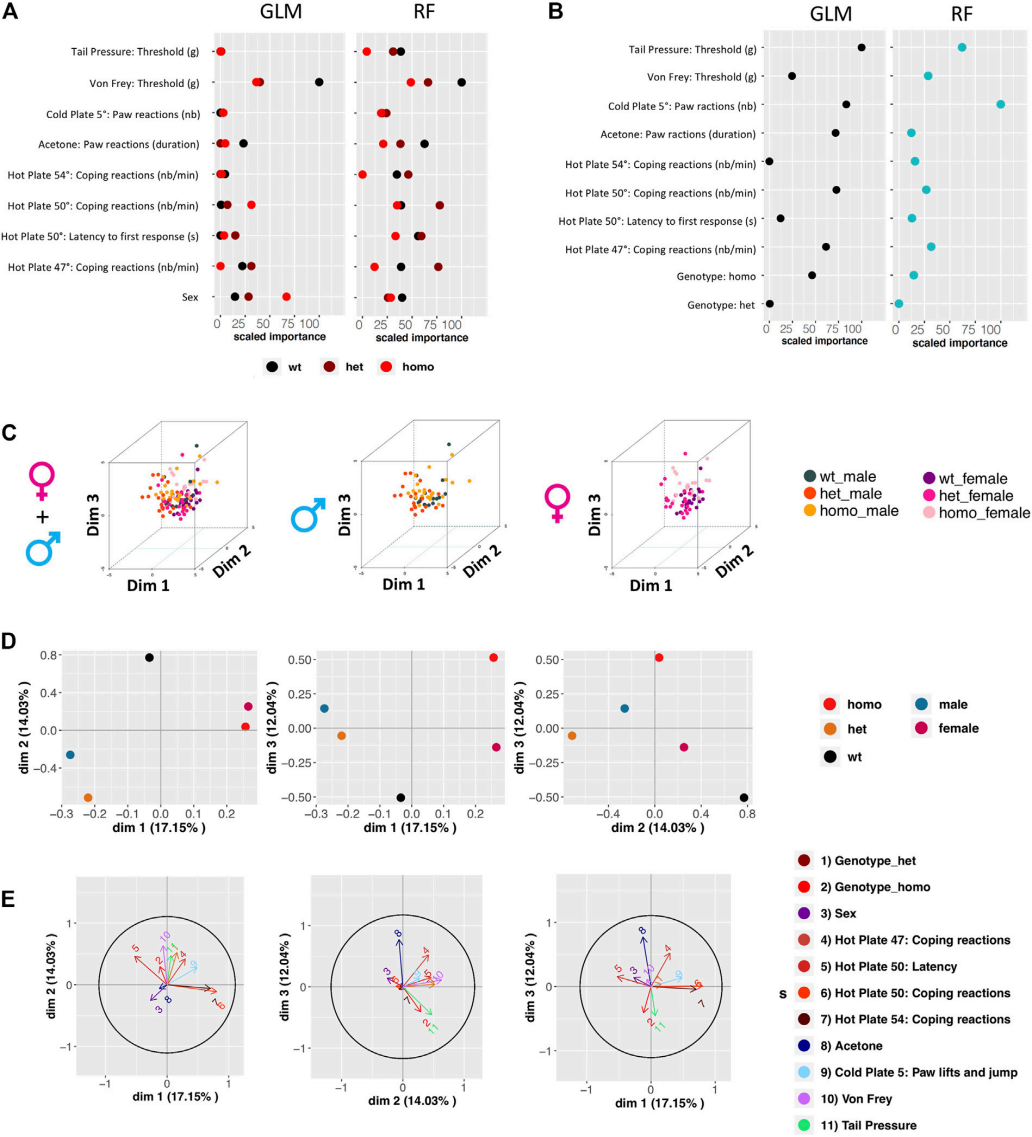
Gdaphen analyses to identify the variables contributing more to genotype and sex discrimination considering the three genotypes together, based on phenotypic data. **(A)** Identification of the power of each explanatory phenotypic variable to the genotype discrimination. The explanatory variables selected were the ones known to contribute more than a 30% to the genotype discrimination. Those variables were identified using a principal component analysis (PCA) after applying a multifactor analysis of mixed data (MFA) implemented using MFAmix function from the PCAmixdata R package (https://arxiv.org/abs/1411.4911). The relevance of those variables to the genotype discrimination was analyzed using two different statistical classifiers: Generalized linear models, noted as GLM taken from the caret R package, and Random forest, noted RF taken from the caret R package (Max Kuhn (2020). Caret: Classification and Regression Training. R package version 6.0-86. https://CRAN.R-project.org/package=caret)). All measures of importance are scaled to have a maximum value of 100 in the variable contributing the most to the discrimination. With GLM and RF, we can identify the importance of each variable to discriminate each genotype. **(B)** Identification of the power of each explanatory phenotypic variable known to contribute more than a 30% to the genotype discrimination, analyzed to see the existence of different responses based on the sex using the two different statistical classifiers. All measures of importance are scaled to have a maximum value of 100 in the variable contributing the most to the discrimination. **(C)** 3D-PCA plots showing the individual animals clustering on the 3D space based on the PCA analyses performed with all the phenotypic variables and colored based on genotype and sex. On the left panel showing the three genotypes and two-sex individuals, on the middle plot showing only male data, and on the right plot only female data. **(D)** Qualitative variable discrimination component map. Showing the distribution in 2D space of the qualitative variable coordinates calculated based on the PCA analysis performed after the MFA implemented using MFAmix function from the PCAmixdata R package. **(E)** Identification of correlation between each principal component of the separated analyses of each group of variables and the principal components (dimensions) calculated by the MFAmix function. By the arrow length, we could identify the contribution of each grouped variable to the discrimination on each dimension. Arrows that follow similar trajectories contribute to the separation of the individual data in the same dimensions.

We sought to identify which variables contribute most to genotype and sex discrimination, by assessing how well each variable could discriminate between the three genotypes (wt, heterozygous, and homozygous) and between the two sexes. By performing the Gdaphen analysis, we considered 14 variables in total, genotype, sex, and 12 behavioral variables (see Supplementary Method). Among these 14, 9 variables were detected as the most relevant (>30%) to discriminate between the three mouse genotypes. These variables are sex, coping reactions on the 47°C and 54°C hot plate, coping reactions and latency on the 50°C hot plate, acetone test, paw lifts and jumps on a 5°C cold plate, von Frey, and tail pressure. Generalized linear model (GLM) (Figure 5A and Supplementary Figure S2.1A) and random forest (RF) (Figure 5A and Supplementary Figure S2.1B) classifiers identified von Frey as the most important variable discriminating between the three genotypes. The RF analysis also identified acetone, coping reactions, and latency on the 50°C hot plate and tail pressure as major explanatory variables. For sex discrimination, GLM (Figure 5B and Supplementary Figure S2.2A) and RF (Figure 5B and Supplementary Figure S2.2B) identified tail pressure, paw lifts and jumps on the 5°C cold plate, and coping reactions on the 50°C hot plate as the most important variables. GLM also identified acetone as an important variable, and RF also identified coping reactions on the 47°C hot plate as an important one. Based on the qualitative variable discrimination 2D component map, sex had a stronger effect over genotype for discrimination between groups. Indeed, heterozygous and homozygous animals are more strongly separated from wt in dimensions 2 and 3, than in dimension 1; males and females are highly separated in dimension 1, but not in the other dimensions (Figure 5D). The multifactor analysis of mixed data (MFA) was used to assess the importance of each test after grouping the variables recorded in each test. By examining the correlation between the location of each grouped variable on the principal components, we show that von Frey and coping reactions on the 47°C hot plate contribute the most to genotype discrimination in dimensions 1 and 2 and dimensions 2 and 3. Tail pressure was also identified in dimensions 1 and 2 and paw lifts and jumps on the 5°C cold plate in dimensions 2 and 3. Coping reactions on the 50°C and 54°C hot plate and paw lifts and jumps on the 5°C cold plate were found to contribute the most to genotype discrimination in dimensions 1 and 3, with the strongest contribution in dimension 1 (Figure 5E).

## Discussion

The SCN10A^G1662S^ mutation was described in three patients with painful SFN. We have generated a mouse model harboring this mutation using homologous recombination in ES cells and characterized this mouse line to understand the relationship between this mutation and the pain phenotype on the patients. The Scn10a mRNA is known to be expressed in DRGs that contain cell bodies of sensory neurons; thus, we studied the impact of the mutation on Scn10a transcript expression in DRGs. The Scn10a^G1663S^ mutation did not alter the Scn10a transcript expression level in DRG in mutant mice of both sexes. IENFD was found unchanged in hindpaw skin of G1663S/G1663S mice. Finally, we investigated the sensitivity to mechanical, heat, and cold stimuli of the mutants in both sexes. Our results collectively indicate that the Scn10a^G1663S^ mutant mice have an enhanced pain sensitivity toward mechanical, heat, and cold stimuli in some behavioral tests, which correlate with the pain symptoms of the SFN patients carrying the same mutation.

### Normal IENFD in the Scn10a^G1663S^ Mice

IENFD was normal in Scn10a^G1663S^ in adult mutant mice, similarly to the SFN patients with SCN10A^G1662S^ mutation that had normal IENFD values compared to age-matched and gender-matched normative values. However, patients carrying other point mutations in SCN10A have shown a loss of IENFs; thus, the loss of IENFs appears to be mutation dependent. A father–son pair harboring L554P mutation in the SCN10A gene has a complete depletion of IENFs and severe reduction in density of dermal nerve bundles, which appeared fragmented due to axonal degeneration (Faber et al., 2012; Faber et al., 1944). Other SFN patients with I1706V and D1639N mutations in the SCN10A gene also showed a remarkably decreased IENFD (Huang et al., 2013; Dabby et al., 2016). Indeed, the study by Bianca de Greef et al. indicated that, out of 921 patients with SFN, 614 had normal IENFD with abnormal thermal thresholds (de Greef et al., 2018), indicating that abnormal IENFD is a secondary criterion for painful SFN. The normal IENFD found in Scn10a^G1663S^ mutant mice therefore suggests that in mice also, Scn10a mutants can show normal IENFD and abnormal pain responses.

### Enhanced Sensitivity to Mechanical Stimuli in the Scn10a^G1663S^ Mice

The enhanced sensitivity to mechanical stimuli in the Scn10a^G1663S^ mice can be compared to other genetic mouse models for Scn10a. The Possum mice harboring a Scn10a^T790A^ gain-of-function mutation were identified in an N-ethyl-N-nitrosourea-induced mutagenesis screen. Amanda Blasius and others reported abnormal mechanical behavior in Possum mice. Homozygous Possum mice showed no alteration in sensitivity to touch stimuli in the von Frey test while they showed increased sensitivity when a needle prick was applied to hindpaw (Blasius et al., 2011; Garrison et al., 2014). The Scn10a knockout (KO) mouse model generated by Akopian and others (Akopian et al., 1999) was studied for pain behaviors in many studies (see (Xue et al., 2021) for a recent review). The KO of the Scn10a gene did not impact on touch stimuli as assessed by the von Frey test (Akopian et al., 1999; Kerr et al., 2001; Laird et al., 2002; Nassar et al., 2005; Leo et al., 2010; Shields et al., 2012; Minett et al., 2014a) while it showed reduced pain sensitivity toward noxious mechanical stimuli as assessed by the tail pressure test (Akopian et al., 1999; Nassar et al., 2005; Minett et al., 2014b). Those findings suggested that the presence of Scn10a is important for sensitivity to noxious mechanical stimuli, but not to light touch. A third mouse model was developed where the Cre gene was knocked into Scn10a exon 1, the Scn10a^Cre/+^ hemizygous mice. These mice had no alteration in response to von Frey filaments (Nassar et al., 2005; Stirling et al., 2005), which is in agreement with the results obtained with Scn10a KO mice. In addition, these mice behaved similarly to their wt littermates in the tail pressure test (Agarwal et al., 2004; Stirling et al., 2005), showing that inactivation of one Scn10a allele does not impair mechanical sensitivity. Our results show a reduced mechanical threshold of the Scn10a^G1663S^ mice in the von Frey test. A phenotype in this test has not been previously reported in the other Scn10a mutant lines (Akopian et al., 1999; Kerr et al., 2001; Laird et al., 2002; Agarwal et al., 2004; Nassar et al., 2004; Nassar et al., 2005; Stirling et al., 2005; Leo et al., 2010; Blasius et al., 2011; Shields et al., 2012; Minett et al., 2014a; Garrison et al., 2014) which displayed altered sensitivities to noxious mechanical stimuli (Akopian et al., 1999; Minett et al., 2014b). With the help of Gdaphen analysis, we identified sensitivity to touch (von Frey filaments) as the most important phenotype contributing to increased pain sensitivity in Scn10a^G1663S^ mice. The increased sensitivity to touch in the mutant mice concurs with the SCN10A^+/G1662S^ SFN patient reports on sheet intolerance. The increase in touch sensitivity in Scn10a^G1663S^ mice is also an interesting finding for preclinical research on pain as the von Frey test is the most used test for assessing touch sensitivity in experimentally induced pain models in rodents. The higher touch sensitivity in mutant mice is consistent with the mechanical allodynia that is usually observed in preclinical models of neuropathic pain and could be interpreted to suggest an increase in the excitability of mechanical nociceptors.

### Sensitivity to Cooling and Cold Stimuli in the Scn10a^G1663S^ Mice

Previous works reported that Scn10a mutant mice show alterations in the response to extreme cold as assayed by cold plate behaviors in Scn10a mutant mice. The Possum Scn10a^T790A^ mice showed increased reactions on the cold plate at −1°C (Blasius et al., 2011). Collectively, previous studies with Cold Plate and acetone tests on Scn10a KO mice showed that Scn10a loss of function induces a loss of sensitivity to extreme cold but not to cool and mild cold (Zimmermann et al., 2007; Minett et al., 2012; Minett et al., 2014a; Luiz et al., 2019). More precisely, Scn10a KO mice were less sensitive than wt when nocifensive responses were measured on the cold plate at 0°C (Zimmermann et al., 2007). Scn10a^Cre/Cre^ mice presented a reduced pain phenotype by jumping later than wt mice on the −5°C cold plate and no phenotype when forepaw lifting on the cold plate was assessed at 10°C and 5°C (Luiz et al., 2019). Our study shows no difference in the mean number of hindpaw lifts and total jumps on the cold plate at 5°C. In the future, further investigations using colder temperatures on the plate may reveal an influence of the Scn10a^G1663S^ mutation on sensitivity to extreme cold as already shown in previous studies on other Scn10a mouse models (Zimmermann et al., 2007; Blasius et al., 2011; Luiz et al., 2019). Our findings in the acetone test suggest that the increase in Scn10a excitability induced by the G1663S mutation (Huang et al., 2013; Han et al., 2014) alters the sensitivity to cooling while the loss of function of Scn10a, investigated with the Scn10a KO mice, did not impact this sensitivity (Minett et al., 2012; Minett et al., 2014a; Luiz et al., 2019). Interestingly, G1663S/G1663S males did reactions to acetone similar to wt males while G1663S/G1663S females showed a tendency for longer duration of reactions. Thus, sex appears to influence the effect of the mutation on cooling sensitivity. Our results on increased sensitivity to cooling in the mutant females concur with the aggravated complaints of G1662S SFN patients when exposed to cold and with abnormal thresholds for cold sensation in the QST.

### Sensitivity to Heat Pain in the Scn10a^G1663S^ Mice

In the Hargreaves test, the Scn10a KO animals displayed analgesia when a slow ramp for radiant heat was used (13–15 s latency in wt mice) (Akopian et al., 1999; Minett et al., 2014b) while no phenotype was found when using a faster heat ramp (7–8 s latency in wt mice) (Nassar et al., 2005; Leo et al., 2010; Shields et al., 2012), indicating that the use of a slow temperature ramp allowed to detect analgesia in the Scn10a KO mice (Xue et al., 2021). In our study, the G1663S male animals were more sensitive in the Hargreaves test when using a heating ramp that led to a withdrawal latency of 7 s for wt mice similar to the latency described in the fast-ramp papers (Nassar et al., 2005; Leo et al., 2010; Shields et al., 2012). Therefore, in a fast-ramp condition previously reported to lead to no phenotype in the KO mice, we could evidence the increased sensitivity of G1663S male animals to radiant heat applied to the paw. In the tail flick assay, sex-grouped mice showed no phenotype, while mutant females had an overall higher reaction latency compared to control mice. An increased latency was found in Scn10a KO mice in this test under a heating condition leading to a longer latency in wt mice than in the present study, and with mice of unknown sex (Akopian et al., 1999). Also, Scn10a KO mice showed no phenotype in the tail immersion test (Akopian et al., 1999; Nassar et al., 2005; Leo et al., 2010). Altogether, the Scn10a^G1663S^ line did not show increased sensitivity in the tail flick assay in the present experimental condition, and slower heating ramps may be further assayed. The sex difference for the mutation effect in the Hargreaves and tail flick tests could also be explained by sex differences in paw and tail skin innervation.

In the hot plate assay, a shorter latency to the first hindpaw reaction was found for G1663S male mice at 54°C, while no phenotype was found at the lower 47°C and 50°C temperatures. The Possum mice showed no alteration on the hot plate at 52°C (Blasius et al., 2011), and their sensitivity at other temperatures or for other heat tests was not reported. The Scn10a KO mice showed no phenotype in the hot plate assay when the latency to first hindpaw withdrawal reaction was recorded at 45°C, 50°C, 55°C, and 60°C (Akopian et al., 1999; Nassar et al., 2005; Leo et al., 2010). Also, Scn10a^Cre/+^ mice had normal responses in the hot plate test at 50°C and 55°C (Agarwal et al., 2004; Nassar et al., 2005; Stirling et al., 2005).

Altogether, our findings on Scn10a^G1663S^ mice in the three tests for heat sensitivity indicate that the Scn10a^G1663S^ mutation leads to increased sensitivity to strong heating of the paw in males. This is in global agreement with previously published results on mutant mice in which Scn10a has been inactivated (Akopian et al., 1999; Nassar et al., 2005; Leo et al., 2010; Minett et al., 2014b; Xue et al., 2021), although the sex effect was not fully explored in this studies.

### Sex Effect on the Scn10a^G1663S^ Mouse Behaviors

In our study, we assessed pain behaviors of Scn10a^G1663S^ mice on both sexes and analyzed the data of each sex separately and sex-grouped. The results suggest that there are specific sex differences in Scn10a^G1663S^ mutants. Mutant females showed increased reactions in the acetone test while mutant males had an increased sensitivity in the Hargreaves test and on the 54°C hot plate. The sex-grouped analysis allowed to detect an increased sensitivity to mechanical stimuli. This suggests that the effect of the mutation is influenced by the sex. The three SFN patients carrying the SCN10A^G1662S^ mutation were women (Han et al., 2014; Eijkenboom et al., 2019), whose symptoms globally correlate with the pain phenotype found in Scn10a^G1663S^ mutant animals. Nevertheless, no SFN male patients with the SCN10A^G1662S^ mutation have been reported until now. There are multifactorial and complex influences underlying the sex differences in pain in the clinical setting. Pain is reported more frequently by women than men. Healthy women have significantly lower pain thresholds for electrical, pressure, and thermal stimuli (Nasser and Afify, 2019). Sex hormones and sociocultural factors are known to impact men and women pain experience (Nasser and Afify, 2019). Women manifest their pain with stronger emotion and evoke pain distress, while men seem to withstand pain and less eager to report pain (Nasser and Afify, 2019). In addition, pain thresholds of both male and female rodents can be affected by the sex of the examiner (Kállai et al., 2004; Gijsbers and Nicholson, 2005). Also, sex differences in opioid-induced analgesia were reported (Niesters et al., 2010; Mogil, 2020). In future studies, it would be interesting to evaluate further mutant mice of both sexes for their response to inflammation-induced pain and for analgesia induced by opioids, gabapentinoids, and anti-depressants.

In recent years, more studies have used animals of both sexes and reported sex-dependent differences or no differences in nociceptive behaviors in rodent models (Mogil, 2020). In addition, the genetic background may impact on sex differences or no differences in pain. In the last decade, a neuroimmune mediation of pain has also been discovered, with the preferential involvement of spinal microglia in pain hypersensitivity in male rodents (Rosen et al., 2017; Sorge and Totsch, 2017; Mogil, 2020). The sex differences found in behavioral responses in Scn10a^G1663S^ mutants contribute to complete the list of genes and mutations for which a sex effect has been detected.

## Conclusion and Perspectives for Scn10a^G1663S^ Mice

Globally, the studies on Scn10a genetic rodent models have highlighted the important role of the NAV1.8 channel including the SCN10A protein in nociception (Xue et al., 2021). Also, the Nav beta4 subunit has been shown to contribute to sensory neuron excitability *in vitro* (Xiao et al., 2019). Still, rodent models of the human SCN10A mutations that were identified in SFN patients were needed to study those mutations *in vivo*. We have successfully created the mouse model for SCN10A^G1662S^ mutation identified in SFN patients by using homologous recombination in ES cells. Scn10a^G1663S^ mutant mice revealed a pain phenotype toward non-noxious and noxious mechanical stimuli, cooling stimuli, and some forms of heat stimuli. Moreover, by the help of the Gdaphen statistical analysis, we could identify which nociception tests allowed to discriminate the most mutants from wt control animals. Hereafter, other devices could also be used to assess thermal preference as complementary tests for heat sensitivity, such as the two-plate choice test (Bautista et al., 2007), the thermal gradient test (Bohic et al., 2020), or the operant plantar thermal assay (Reker et al., 2020). Our results show that Scn10a^G1663S^ mutant animals concur in pain features with the SFN patients carrying the SCN10A^G1662S^ mutation, such as sensitive skin and abnormal thresholds for warm and cold stimuli as revealed by QST. In the future, spontaneous pain may also be assessed in the mutant mice. Different paradigms could be used to evaluate spontaneous pain, including weight bearing or conditioned place preference for the chamber associated with analgesic administration (Barrot, 2012; Tappe-Theodor et al., 2019).

The development of genetic rodent models recapitulating the SCN mutations found in SFN patients is a key step to explore pain mechanism involving sodium channels and the pillar to develop convenient novel analgesics. The Scn10a^G1663S^ mouse model opens the door for further investigations in the aim of complementing the knowledge about the role of NAV1.8 channel in painful SFN.

## Data Availability

The original contributions presented in the study are included in the article/Supplementary Material; further inquiries can be directed to the corresponding author.
