# Supplementary material for: The Human SCN10AG1662S Point Mutation Established in Mice Impacts on Mechanical, Heat, and Cool Sensitivity
Source: Front Pharmacol. 2021 Dec 1;12:780132. doi: 10.3389/fphar.2021.780132 (PMC8671994; doi:10.3389/fphar.2021.780132)
Supplement: Supplementary file 1 [file DataSheet1.PDF]

The human $SCN10A^{G1662S}$ point mutation established in mice impacts on mechanical, heat and cool sensitivity

Celeste Chidiac, Yaping Xue, Maria del Mar Muniz Moreno, Ameer Abu Bakr Rasheed, Romain Lorentz, Marie-Christine Birling, Claire Gaveriaux-Ruff* and Yann Herault*

Supplementary Materials

Supplementary Method. Gdaphen analysis for the identification of the variables contributing the most to the genotype or sex discrimination

Gdaphen is a R pipeline that allows the identification of the most important predictor qualitative and quantitative variables for genotype discrimination in animal models of different diseases. We used gdaphen an unpublished R package developed by Maria del Mar MUNIZ MORENO in our team (Muniz et al soon to be submitted to CRAN/ available on github https://github.com/munizmom) to identify the explanatory variables from experimental data. The gdaphen R package uses the R packages listed in Supplementary Table 8. The variables included genotype and sex as well as all behavioral data obtained during this project. The aim was to identity the most relevant variables that contributed to the discrimination between the three mouse genotypes: wt, *Scn10a*$^{+/G1663S}$, *Scn10a*$^{G1663S/G1663S}$.

Gdaphen principle

Gdaphen takes as input data an excel table containing on the rows the info per animal and on each column all the variables recorded. As several variables were recorded for some tests, we grouped those variables with the same group labeled test name and identified the importance for the discrimination of i) each variable alone, ii) the overall contribution of the group or test. In order to test the contribution of each variable alone, all variables had the same weight in the analysis.

Instead, for testing the overall contribution of each test after grouping the parameters that were recorded in the same test, we performed a multifactor analysis of mixed data (MFA) that provides a weight to each variable to avoid giving more importance to tests with a higher number of parameters tested, see part 3 on MFA analysis.

Then, some pre-processing steps are necessary to get the data into shape for the analysis:

1) **Pre-processsing steps:**

- **Imputation of NAs if they exist:**

  If one missing value exist per genotype/sex/variable and the number of animals is >10 then the imputed value is the calculated mean of the values for that genotype/sex/variable.

- Instead if more than one missing value exists per genotype/sex/variable and the total number of missing values are one per 10 animals then we implemented a method for imputation using Additive Regression, Bootstrapping, and Predictive Mean Matching based in closest random sampling implemented over the aregImpute function from the Hmisc R package (Frank E Harrell Jr, with contributions from Charles Dupont and many others. (2021). Hmisc: Harrell Miscellaneous. R package version 4.5-0. https://CRAN.R-project.org/package=Hmisc).
- **Removal of quantitative variables with less than 3 different unique values** as no error or standard deviation can be calculated with so little different numbers.
- **Removal of qualitative variables** with a unique categorical value as we don't have any possible discrimination
- **Standardization of the data** by scaling: this step is necessary as each independent variable has a different range of values that are observed and is necessary to calculate the contribution of each variable in a comparable way by re-scaling each variable so all have the same range of values observed and/or the same variance.

2) **Identification of the variables contributing more to the discrimination of genotype or sex by using classifiers.**

These classifiers are algorithms that will assign the data into one of the possible set of "classes" or categories previously defined. We decided to use two different classifiers to give answer to two different questions:

- A supervised algorithm that will allow us to identify which phenotypic variables or "predicting variables", if they have an exponential family distribution, are able to discriminate due to the fact that their linear combination is influencing the value of the dependent variable response.

1) If the dependent variable to be discriminated have two factors (for example the variable sex, has two possible categories "male" or "female"), or more than two (for example the variable genotype, has three possible categories), we use a Generalized Linear Model, noted as GLM, from the caret R package. (Max Kuhn (2020). caret: Classification and Regression Training. R package version 6.0-86. https://CRAN.R-project.org/package=caret). GLM can identify the contribution of each phenotypic variable on the discrimination of each specific category of the dependent variable.
2) An unsupervised algorithm that will be able to identify relevant phenotypic variables for the discrimination even though there may not be coming from a linear distribution or exponential distribution family. We decided to implement the Random Forest algorithm, noted as RF, from the caret R package. (Max Kuhn (2020). caret: Classification and Regression Training. R package version 6.0-86. https://CRAN.R-project.org/package=caret). This classifier builds a forest of 100000 individual decision trees per observation and predict in ensemble the class of the outcome (or the category of the dependent variable). Is based on the ***wisdom of the crowds*** principle, as a large number of relatively uncorrelated models (trees) as a team will outperform any individual tree decision.

3) Identify the weight of each test to the prediction and visualization using the Multiple Factor Analysis (MFA).

This method was used to analyze groups of variables both qualitative and quantitative recorded from the same individuals. The MFA analysis was used to assess the importance of each test after grouping the variables recorded in each test. The MFA was developed by Escofier and Pages in 1994 (Multiple factor analysis (afmult package). Computational statistics & data analysis, 18(1):121-140) and by Abdi et al. in 2013 (Abdi H., Williams L., Valentin D. (2013) ; Multiple factor analysis: principal component analysis for multitable and multiblock data sets. https://doi.org/10.1002/wics.1246) and was implemented by Chavent et al. in 2017 (see in Suppl. Table 8). It performs a normalization or "weighting" on each group. The "weighting"

is necessary to be able to assess the importance of the grouped variables without giving more importance to groups with a higher number of parameters tested. The weight was calculated by dividing all the variables belonging to the group by the first eigenvalue coming from the principal component analysis (PCA) of the group. Then a PCA on all the weighted variables is applied and we can identify the correlation between the qualitative or quantitative variables grouped or ungrouped, and the principal component dimensions or identify the individual coordinates of each observation on the PCA dimensions. The method is implemented using the MFAmix function from the PCAmixdata R package (Frank E Harrell Jr, with contributions from Charles Dupont and many others. (2021). Hmisc: Harrell Miscellaneous. R package version 4.5-0. https://CRAN.R-project.org/package=Hmisc).

a. **Pre-selection of phenotypic variables for the analysis to increase the variance explained of the data using those selected variables.**

We analysed three different number of phenotypic predictor variables.

i) All phenotypic variables

ii) The phenotypic variables left after removing the highly correlated ones (correlation higher than 75%).

iii) The phenotypic variables contributing in the discrimination more than a 30% after running the MFA analysis using all variables and observing the correlation between the quantitative ungrouped phenotypic variables with the main three dimensions of the PCA. Our reasoning is to try to decrease the noise added by variables that are not strongly contributing to the discrimination, decrease the complexity of the model and the calculations and increase the power on the discrimination as lower number of variables are considered. To assure we are not performing worse with this model than with the model created using all the variables, we calculated the variance of the data we are able to explain using the first 10 dimensions and the accuracy of the models to answer to how well they can predict correctly each individual observation to the class of the dependent variable.

3) We run gdaphen pipeline to perform the genotype and sex discrimination analyses on all genotypes: *Scn10a*$^{+/G1663S}$, *Scn10a*$^{G1663S/G1663S}$ and control littermates' phenotypic data.

```
Human MEFPIGSLETNNFRRFTPESLVEIEKQIAAKQGTKKAREKHREQKDQEEKPRPQLDLKACNQLPKFYGELP
      |||| ||  | |||||||||| ||||||||    || | | | |||  ||||||||||||||| ||||||
Mouse MEFPFGSVGTTNFRRFTPESLAEIEKQIAAHRAAKKGRPKQRGQKDKSEKPRPQLDLKACNQLPRFYGELP

Human AELIGEPLEDLDPFYSTHRTFMVLNKGRTISRFSATRALWLFSPFNLIRRTAIKVSVHSWFSLFITVTILV
      ||| ||||||||||||||||| || | ||||||||| ||||||||||||||||||||||||||||||||||
Mouse AELVGEPLEDLDPFYSTHRTFIVLDKSRTISRFSATWALWLFSPFNLIRRTAIKVSVHSWFSIFITVTILV

Human NCVCMTRTDLPEKIEYVFTVIYTFEALIKILARGFCLNEFTYLRDPWNWLDFSVITLAYVGTAIDLRGISG
      ||||||||||||| || ||| |||||||||||||||||||||||||||||||||||||||| |||||||||
Mouse NCVCMTRTDLPEKLEYAFTVVYTFEALIKILARGFCLNEFTYLRDPWNWLDFSVITLAYVGAAIDLRGISG

Human LRTFRVLRALKTVSVIPGLKVIVGALIHSVKKLADVTILTIFCLSVFALVGLQLFKGNLKNKCVKNDMAVN
      |||||||||||||||||||||||||||||| ||||||||| |||||||||||||||||||||| ||
Mouse LRTFRVLRALKTVSVIPGLKVIVGALIHSVRKLADVTILTVFCLSVFALVGLQLFKGNLKNKCIKNGTDPH
                     300
Human ETTNYSSHRKPDIYINKRGTSDPLLCGNGSDSGHCPDGYICLKTSDNPDFNYTSFDSFAWAFLSLFRLMTQ
         | ||    || | | || |||||||||| ||||  | | |||||||||||||||||||||||||||||
Mouse KADNLSSEMAGDIFI-KPGTTDPLLCGNGSDAGHCPNDYVCRKTSDNPDFNYTSFDSFAWAFLSLFRLMTQ

Human DSWERLYQQTLRTSGKIYMIFFVLVIFLGSFYLVNLILAVVTMAYEEQNQATTDEIEAKEKKFQEALEMLR
      |||||||||||| ||| || |||||||||||||||||||||||||||| |||  ||||||||| |||| |
Mouse DSWERLYQQTLRASGKMYMVFFVLVIFLGSFYLVNLILAVVTMAYEEQSQATIAEIEAKEKKFKEALEVLQ

Human KEQEVLAALGIDTTSLHSHNGSPLTSKNASERRHRIKPRVSEGSTEDNKSPRSDPYNQRRMSFLGLASGKR
      |||||||||||||||| |||||||  ||| ||| | | | ||||| || |  |||||||||||||| || |
Mouse KEQEVLAALGIDTTSLYSHNGSPLAPKNANERRPRVKSRMSEGSTDDNRSLQSDPYNQRRMSFLGLSSGRR

Human RASHGSVFHFRSPGRDISLPEGVTDDGVFPGDHESHRGSLLLGGGAGQQGPLPRSPLPQPSNPDSRHGEDE
      |||| |||||| |  | | | |  ||||| || || | | ||| |||| ||||||||||  ||  | ||
Mouse RASHSSVFHFRAPSQDVSFPDGILDDGVFHGDQESRRSSILLGRGAGQAGPLPRSPLPQSPNPGPRRGEEG

Human HQPPPTSELAPGAVDVSAFDAGQKKTFLSAEYLDEPFRAQRAMSVVSIITSVLEELEESEQKCPPCLTSLS
          || ||| ||    | ||   | |||| || |||||||||||||| ||| ||||||  |||||| ||
Mouse QRGVPTGELATGAPEGPALDAAGQKNFLSADYLNEPFRAQRAMSVVSIMTSVIEELEESKLKCPPCLISLA

Human QKYLIWDCCPMWVKLKTILFGLVTDPFAELTITLCIVVNTIFMAMEHHGMSPTFEAMLQIGNIVFTIFFTA
      |||||| ||| | | |  || ||||||||||||||||||| ||||||  |   | |||| |||||| |||
Mouse QKYLIWECCPKWKKFKMVLFELVTDPFAELTITLCIVVNTVFMAMEHYPMTDAFDAMLQAGNIVFTVFFTM

Human EMVFKIIAFDPYYYFQKKWNIFDCIIVTVSLLELGVAKKGSLSVLRSFRLLRVFKLAKSWPTLNTLIKIIG
      || ||||||||||||||||||||| |||||||||   ||||||||| ||||||||||||||||| ||||||
Mouse EMAFKIIAFDPYYYFQKKWNIFDCVIVTVSLLELSTSKKGSLSVLRTFRLLRVFKLAKSWPTLNMLIKIIG

Human NSVGALGNLTIILAIIVFVFALVGKQLLGENYRNNRKNISAPHEDWPRWHMHDFFHSFLIVFRILCGEWIE
      |||||||||| ||||||||||||||||| |||   |  ||  ||   |||| ||||||| |||||||||||
Mouse NSVGALGNLTFILAIIVFIFALVGKQLLSENYGCRRDGISVWNGERLRWHMCDFFHSFLVVFRILCGEWIE
```

Supplementary figure 1. Alignment of SCN10A protein sequence in human and mouse species. The Ensemble BLAST alignment shows deletion of one residue and insertion of two residues in mouse sequence as compared to human sequence. The site for 1662/1663 residue is highlighted.

```
Human NMWACMEVGQKSICLILFLTVMVLGNLVVLNLFIALLLNSFSADNLTAPEDDGEVNNLQVALARIQVFGHR
      ||| |||| |  |||||||||||||||||||||||||||||||||||||||||||||||||||||||||||
Mouse NMWVCMEVSQDYICLTLFLTVMVLGNLVVLNLFIALLLNSFSADNLTAPEDDGEVNNLQVALARIQVFGHR
                                                           978-979
Human TKQALCSFFSRSCPFPQPKAEPELVVKLPLSSSKAENHIAANTARGSSGGLQAPR--GPRDEHSDFIANPT
         |  |     |    || |  |  | || | |||||||        | |  |   ||   | |||  |
Mouse ASRAITSYIRSHCRLRWPKVETQLGMKPPLTSCKAENHIATDAVNAAVGNLAKPALGGPKENHGDFITDPN

Human VWVSVPIAEGESDLDDLEDDGGEDAQSFQQEVIPKGQQEQLQQVERCGDHLTPRSPGTGTSSEDLAPSLGE
      ||||||||||||||| || |     ||  ||  |||||| ||||  | ||   |||  | ||||||| |||
Mouse VWVSVPIAEGESDLDELEEDVEHASQSSWQEESPKGQQELLQQVQKCEDHQAARSPPSGMSSEDLAPYLGE

Human TWKDESVPQVPAEGVDDTSSSEGSTVDCLDPEEILRKIPELADDLEEPDDCFTEGCIRHCPCCKLDTTKSP
       |  |  | ||||||||||||||||||| |||||||||||||  | |||||| ||| | |||||  | | |
Mouse RWQREESPRVPAEGVDDTSSSEGSTVDCPDPEEILRKIPELAEELDEPDDCFPEGCTRRCPCCKVNTSKFP

Human WDVGWQVRKTCYRIVEHSWFESFIIFMILLSSGSLAFEDYYLDQKPTVKALLEYTDRVFTFIFVFEMLLKW
      |  |||||||||||||||||||||||||||||| ||||| ||  || ||  ||||||||||||||||||||
Mouse WATGWQVRKTCYRIVEHSWFESFIIFMILLSSGALAFEDNYLEEKPRVKSVLEYTDRVFTFIFVFEMLLKW

Human VAYGFKKYFTNAWCWLDFLIVNISLISLTAKILEYSEVAPIKALRTLRALRPLRALSRFEGMRVVVDALVG
      ||||||||||||||||||||||||| || ||||||| || |||||||||||||||||||||||||||||||
Mouse VAYGFKKYFTNAWCWLDFLIVNISLTSLIAKILEYSDVASIKALRTLRALRPLRALSRFEGMRVVVDALVG

Human AIPSIMNVLLVCLIFWLIFSIMGVNLFAGKFWRCINYTDGEFSLVPLSIVNNKSDCKIQNSTGSFFWVNVK
      ||||||||||||||||||||||||||||||| ||       || |    | |||||  || || |||||||
Mouse AIPSIMNVLLVCLIFWLIFSIMGVNLFAGKFSRCVDTRSNPFSVVNSTFVTNKSDCYNQNNTGHFFWVNVK

Human VNFDNVAMGYLALLQVATFKGWMDIMYAAVDSREVNMQPKWEDNVYMYLYFVIFIIFGGFFTLNLFVGVII
      |||||||||||||||||||||||||||||||||  | || ||   ||||||| ||||||||||||||||||
Mouse VNFDNVAMGYLALLQVATFKGWMDIMYAAVDSRDINSQPNWEESLYMYLYFVVFIIFGGFFTLNLFVGVII

Human DNFNQQKKKLGGQDIFMTEEQKKYYNAMKKLGSKKPQKPIPRPLNKFQGFVFDIVTRQAFDITIMVLICLN
      |||||||||  ||||||||||||||||||||||||||||||||||| |||||||||||||||||| |||||
Mouse DNFNQQKKKIRGQDIFMTEEQKKYYNAMKKLGSKKPQKPIPRPLNKYQGFVFDIVTRQAFDIIIMALICLN

Human QSEEKTKILGKINQFFVAVFTGECVMKMFALRQYYFTNGWNVFDFIVVVLSIASLIFSAILKSLQSYFSPT
      ||||||| || ||||||||||||||||||||||||||||||||||||| ||| || ||||| || | ||||
Mouse QSEEKTKVLGRINQFFVAVFTGECVMKMFALRQYYFTNGWNVFDFIVVILSISSLLFSAILSSLESYFSPT

Human LFRVIRLARIGRILRLIRAAKGIRTLLFALMMSLPALFNIGLLLFLVMFIYSIFGMSSFPHVRWEAGIDDM
      | |||||||||||||||||||||||||||||||||||||||||||||||||||||| ||  |  |||||||
Mouse LLRVIRLARIGRILRLIRAAKGIRTLLFALMMSLPALFNIGLLLFLVMFIYSIFGMASFANVIDEAGIDDM
                      1662-1663
Human FNFQTFANSMLCLFQITTSAGWDGLLSP
      ||| || |||||||||||||||||||||
Mouse FNFKTFGNSMLCLFQITTSAGWDGLLSP
```

Supplementary figure 1 (continued)

1 Genotype discrimination

A GLM

Sel model >30%: 9 Variables<br>Accuracy: GLM: 0.38			
wt	het	homo	Variable
12.14	23	49.48	Sex:: Sex_male
100	45	40.66	Von Frey:: Threshold (g)
0	0.42	0.18	Tail Pressure:: Threshold (g)
13.96	0	3.16	Acetone:: Withdrawal and Flicks (duration s)
0	1.66	1.91	Cold Plate 5: Paw lifts and jumps:: Frequency (nb)
12.11	19.79	0	Hot Plate 47: Coping reactions:: Total (nb/min)
0	8.88	2.45	Hot Plate 50: Latency:: First response (s)
0.68	4.35	19.37	Hot Plate 50: Coping reactions:: Total (nb/min)
3.12	0	0.83	Hot Plate 54: Coping reactions:: Total (nb/min)

B RF

Sel model >30%: 9 Variables<br>Accuracy: RF: 0.39			
wt	het	homo	Variable
31.19	27.33	28.4	Sex:: Sex_male
100	66.44	33.76	Von Frey:: Threshold (g)
37.33	18.75	43.99	Tail Pressure:: Threshold (g)
81.95	44.96	37.68	Acetone:: Withdrawal and Flicks (duration s)
30.83	28.44	15.73	Cold Plate 5: Paw lifts and jumps:: Frequency (nb)
0	60.55	36.56	Hot Plate 47: Coping reactions:: Total (nb/min)
42.99	73.07	52.56	Hot Plate 50: Latency:: First response (s)
26.85	77.01	62.08	Hot Plate 50: Coping reactions:: Total (nb/min)
34.45	55.48	20.35	Hot Plate 54: Coping reactions:: Total (nb/min)

2 Sex discrimination

A GLM

Sel model >30%: 9 Variables<br>Accuracy: GLM: 0.56	
Overall	Variable
0.46	Genotype:: Genotype_b.het
46.31	Genotype:: Genotype_c.homo
24.56	Von Frey:: Threshold (g)
100	Tail Pressure:: Threshold (g)
71.8	Acetone:: Withdrawal and Flicks (duration s)
83.4	Cold Plate 5: Paw lifts and jumps:: Frequency (nb)
61.41	Hot Plate 47: Coping reactions:: Total (nb/min)
12.18	Hot Plate 50: Latency:: First response (s)
72.7	Hot Plate 50: Coping reactions:: Total (nb/min)
0	Hot Plate 54: Coping reactions:: Total (nb/min)

B RF

Sel model >30%: 9 Variables<br>Accuracy: RF: 0.59	
Overall	Variable
0	Genotype:: Genotype_b.het
14.63	Genotype:: Genotype_c.homo
28.7	Von Frey:: Threshold (g)
62.05	Tail Pressure:: Threshold (g)
12.25	Acetone:: Withdrawal and Flicks (duration s)
100	Cold Plate 5: Paw lifts and jumps:: Frequency (nb)
31.71	Hot Plate 47: Coping reactions:: Total (nb/min)
12.82	Hot Plate 50: Latency:: First response (s)
26.89	Hot Plate 50: Coping reactions:: Total (nb/min)
15.84	Hot Plate 54: Coping reactions:: Total (nb/min)

Supplementary Fig. 2. Measuring the explanatory variables importance for genotype and sex discrimination considering the three genotypes together, using different statistical classifiers. 1. Genotype discrimination: The relevance of the selected 9 variables to genotype discrimination was analyzed using two different statistical classifiers: **A)** Generalized Linear Models, noted as GLM taken from the caret R package (Max Kuhn (2020). caret: Classification and Regression Training. R package version 6.0-86. https://CRAN.R-project.org/package=caret) and **B)** Random forest, noted RF taken from the caret R package. **2.** Sex discrimination: The relevance of the selected 9 variables to sex discrimination was analyzed using two different statistical classifiers: **A)** GLM and **B)** RF.

Supplementary Table 1A. PCR Primers sequence used for genotyping

Primers position	Primers sequence
Ef	CCAGCTGAACTTGGCTATGGAAGAG
Ef2	GCTTGTAGATGAAGAAGGCAGGG
Er	GTGGGTGAAACAGCCACATGG
Er2	CCGTTCAGTAGCTGTCCACTGC
Er3	CCATCCCTTCCTTGGGTGGTG
Lxr	GAAGTTATACTAGAGCGGCCGTTCAC
Mf	CGGCCTCCTCCTCTTCCTCG
Mr	GGATGCCCACCGCTGGG
Mq1f	CCGCCCATTCTCCGCCC
Mq1r	TGCTAAAGCGCATGCTCCAGACTGC

Supplementary Table 1B. PCR reactions used for genotyping with the corresponding bands size

PCR	Region analyzed	Primer	Recombinant allele	PM allele	WT allele
PCR 1	Excision of selection marker	Ef / Er2	4484*	363	250
PCR 2	Excision of selection marker 2	Ef2 / Er3	4357*	236	123
PCR 3	5’ part of selection marker	Ef / Mq1r	286	-	-
PCR 4	3’ part of selection marker	Mq1f / Er	461	-	-
PCR 5	LoxP specific PCR	Ef / Lxr	194	194	-

Supplementary Table 2. Sequence of *Scn10a* and *Hprt* probes and primers used for ddPCR

Scn10a^{G1663S}-Forward Primer sequence (5'-3')	TCGACTTCATTGTGGTGATTCT
Scn10a^{G1663S}-Reverse Primer sequence (5'-3')	GGTCCTGTGTTGAGGATGG
Scn10a^{+} Probe	/5HEX/ACGTCGGCT/ZEN/GGCTGGGATGG/3IABkFQ/
Scn10a^{G1663S} Probe	/56-FAM/ACGTCGGCT/ZEN/AGCTGGGATGG/3IABkFQ/
Hprt-Forward primer sequence (5'-3')	CCCCAAAATGGTTAAGGTTGC
Hprt -Reverse primer sequence (5'-3')	AACAAAGTCTGGCCTGTATCC
Hprt –Probe	5HEX/CTTGCTGGT/ZEN/GAAAAGGACCTCTCGAA/3IABkFQ/

Supplementary Table 3. ***Scn10a*** **transcript expression in wt and** ***Scn10a**^{G1663S}* **mutant mice**

Gene	Tissue	Alleles	Analysis	Groups	Statistics
Scn10a	DRG	WT	One-way ANOVA for genotype	F & M	***p*<0.0001**<br>F (2, 44) = 144.6
				F	***p*<0.0001**<br>F (2, 21) = 60.3
				M	***p*<0.0001**<br>F (2, 20) = 77.28
			Šídák's multiple comparisons test	F & M +/+ vs +/G1663S	***p*<0.0001**
				F & M +/+ vs G1663S/G1663S	***p*<0.0001**
				F & M +/G1663S vs G1663S/G1663S	***p*<0.0001**
				F +/+ vs F +/G1663S	***p*=0.0001**
				F +/+ vs F G1663S/G1663S	***p*<0.0001**
				F +/G1663S vs F G1663S/G1663S	***p*<0.0001**
				M +/+ vs M +/G1663S	***p*<0.0001**
				M +/+ vs M G1663S/G1663S	***p*<0.0001**
				M +/G1663S vs M G1663S/G1663S	***p*<0.0001**
		PM*	One-way ANOVA for genotype	F & M	***p*<0.0001**<br>F (2, 44) = 245.5
				F	***p*<0.0001**<br>F (2, 21) = 159.7
				M	***p*<0.0001**<br>F (2, 20) = 157.0
			Šídák's multiple comparisons test	F & M +/+ vs +/G1663S	***p*<0.0001**
				F & M +/+ vs G1663S/G1663S	***p*<0.0001**
				F & M +/G1663S vs G1663S/G1663S	***p*<0.0001**
				F +/+ vs F +/G1663S	***p*=0.0001**
				F +/+ vs F G1663S/G1663S	***p*<0.0001**
				F +/G1663S vs F G1663S/G1663S	***p*<0.0001**
				M +/+ vs M +/G1663S	***p*<0.0001**
				M +/+ vs M G1663S/G1663S	***p*<0.0001**
				M +/G1663S vs M G1663S/G1663S	***p*<0.0001**
		Sum of WT + PM	One-way ANOVA for genotype	F & M	*p*=0.221<br>F (2, 44) = 1.56
				F	*p*=0.700<br>F (2, 21) = 0.362
				M	*p*=0.094<br>F (2, 20) = 2.664

* PM, point mutation

Supplementary Table 4. IENF quantification in wt and *Scn10a*^{G1663S} mutant mice

Analysis	Groups	Statistics
Two-way ANOVA	Genotype	*p*=0.421<br>F (2, 18) = 0.907
	Sex	*p*=0.934<br>F (2, 18) = 0.0011
One-way ANOVA	F & M	*p*=0.374<br>F (2, 21) = 2.192
Šídák's multiple comparisons test	F & M +/+ vs +/G1663S	*p*=0.842
	F & M +/+ vs G1663S/G1663S	*p*=0.419

Supplementary Table 5. Normal health conditions and proprioception capacities in wt and *Scn10a*^{G1663S} mutant mice

Test	Parameter	Analysis	Groups	Statistics
	Body weight	One-way ANOVA	F	*p*=0.596<br>F (2, 39) = 0.523
			M	*p*=0.883<br>F (2, 32) = 0.124
String test	Latency	Kruskal-Wallis test	F	*p*=0.855
			M	*p*=0.233
Crenelated bar	Latency	Kruskal-Wallis test	F & M	*p*=0.822
			F	*p*=0.820
			M	*p*=0.763
	Number of mistakes	Kruskal-Wallis test	F & M	*p*=0.682
			F	*p*=0.699
			M	*p*=0.710

Supplementary Table 6. Pain sensitivity to mechanical and cold stimuli in wt and *Scn10a*^{G1663S} mutant mice

Test	Parameter	Analysis	Groups	Statistics
Von Frey	Threshold	One-way ANOVA	F & M	***p*=0.034**<br>F (2, 96) = 3.490
			F	*p*=0.216<br>F (2, 49) = 1.58
			M	*p*=0.157<br>F (2, 44) = 1.930
		Šídák's multiple comparisons test	F & M +/+ vs +/G1663S	*p*=0.082
			F & M +/+ vs G1663S/G1663S	*p*=0.058
		Unpaired t-test two-tailed	F & M +/+ vs +/G1663S	***p*=0.03**
			F & M +/+ vs G1663S/G1663S	***p*=0.025**
Tail pressure	Threshold	Kruskal-Wallis test	F & M	*p*=0.097
			F	*p*=0.123
			M	*p*=0.748
Acetone	Duration withdrawal and flicking	Kruskal-Wallis test	F & M	*p*=0.274
			F	*p*=0.087
			M	*p*=0.603
Cold Plate	Number of paw lifts and jumps	Kruskal-Wallis test	F & M	*p*=0.738
			F	*p*=0.206
			M	*p*=0.551

Supplementary Table 7. Pain sensitivity to heat stimuli in wt and $Scn10a^{G1663S}$ mutant mice

Test	Parameter	T°	Analysis	Groups	Statistics
Hargreaves	Latency		One-way ANOVA	F & M	*p*=0.577<br>F (2, 115) = 0.553
				F	*p*=0.442<br>F (2, 55) = 0.827
				M	***p*=0.047**<br>F (2, 57) = 3.237
			Šídák's multiple comparisons test	M +/+ vs M +/G1663S	*p*=0.273
				M +/+ vs M G1663S/G1663S	***p*=0.043**
			Unpaired t-test two-tailed	M +/+ vs M +/G1663S	*p*=0.139
				M +/+ vs M G1663S/G1663S	***p*=0.018**
Tail flick	Latency		One-way ANOVA	F & M	*p*=0.246<br>F (2, 111) = 1.422
				F	***p*=0.023**<br>F (2, 55) = 4.049
				M	*p*=0.401<br>F (2, 53) = 0.930
			Šídák's multiple comparisons test	F +/+ vs F +/G1663S	*p*=0.729
				F +/+ vs F G1663S/G1663S	*p*=0.127
			Unpaired t-test two-tailed	F +/+ vs F +/G1663S	*p*=0.412
				F +/+ vs F G1663S/G1663S	*p*=0.073
Hot Plate	Latency 1st reaction	47°C	Kruskal-Wallis test	F & M	*p*=0.293
				F	*p*=0.587
				M	*p*=0.308
		50°C	One-way ANOVA	F & M	*p*=0.148<br>F (2, 111) = 1.943
				F	*p*=0.109<br>F (2, 56) = 2.309
				M	*p*=0.918<br>F (2, 52) = 0.086
		54°C	Kruskal-Wallis test	F & M	*p*=0.141
				F	*p*=0.643
				M	***p*=0.049**
			Dunn's multiple comparisons test	M +/+ vs M +/G1663S	*p*>0.999
				M +/+ vs M G1663S/G1663S	*p*=0.266
			Mann Whitney test two-tailed	M +/+ vs M +/G1663S	*p*=0.604
				M +/+ vs M G1663S/G1663S	*p*=0.087
Hot plate	Number of coping reactions	47°C	Kruskal-Wallis test	F & M	*p*=0.077
				F	*p*=0.429
				M	*p*=0.130
		50°C	One-way ANOVA	F & M	*p*=0.277<br>F (2, 111) = 1.3
				F	*p*=0.824<br>F (2, 56) = 0.194
				M	*p*=0.221<br>F (2, 52) = 1.55
		54°C	Kruskal-Wallis test	F & M	*p*=0.626
				F	*p*=0.883
				M	*p*=0.376

Supplementary Table 8. R packages used in Gdaphen

Package	R citation
R	R Core Team (2021). R: A language and environment for statistical computing. R Foundation for Statistical Computing, Vienna, Austria. URL https://www.R-project.org/.
PCAmixdata	Marie Chavent, Vanessa Kuentz, Amaury Labenne, Benoit Liquet and Jerome Saracco (2017). PCAmixdata: Multivariate Analysis of Mixed Data. R package version 3.1. https://CRAN.R-project.org/package=PCAmixdata. https://arxiv.org/abs/1411.4911
caret	Max Kuhn (2020). caret: Classification and Regression Training. R package version 6.0-86. https://CRAN.R-project.org/package=caret
Hmisc	Frank E Harrell Jr, with contributions from Charles Dupont and many others. (2021). Hmisc: Harrell Miscellaneous. R package version 4.5-0. https://CRAN.R-project.org/package=Hmisc
readxl	Hadley Wickham and Jennifer Bryan (2019). readxl: Read Excel Files. R package version 1.3.1. https://CRAN.R-project.org/package=readxl
xlsx	Adrian Dragulescu and Cole Arendt (2020). xlsx: Read, Write, Format Excel 2007 and Excel 97/2000/XP/2003 Files. R package version 0.6.5. https://CRAN.R-project.org/package=xlsx
openxlsx	Philipp Schauberger and Alexander Walker (2020). openxlsx: Read, Write and Edit xlsx Files. R package version 4.2.3. https://CRAN.R-project.org/package=openxlsx
tidyr	Hadley Wickham (2021). tidyr: Tidy Messy Data. R package version 1.1.3. https://CRAN.R-project.org/package=tidyr
dplyr	Hadley Wickham, Romain François, Lionel Henry and Kirill Müller (2021). dplyr: A Grammar of Data Manipulation. R package version 1.0.6. https://CRAN.R-project.org/package=dplyr
gtools	Gregory R. Warnes, Ben Bolker and Thomas Lumley (2020). gtools: Various R Programming Tools. R package version 3.8.2. https://CRAN.R-project.org/package=gtools
gdata	Gregory R. Warnes, Ben Bolker, Gregor Gorjanc, Gabor Grothendieck, Ales Korosec, Thomas Lumley, Don MacQueen, Arni Magnusson, Jim Rogers and others (2017). gdata: Various R Programming Tools for Data Manipulation. R package version 2.18.0. https://CRAN.R-project.org/package=gdata
ggplot2	H. Wickham. ggplot2: Elegant Graphics for Data Analysis. Springer-Verlag New York, 2016.
lattice	Sarkar, Deepayan (2008) Lattice: Multivariate Data Visualization with R. Springer, New York. ISBN 978-0-387-75968-5
mlbench	Newman, D.J. & Hettich, S. & Blake, C.L. & Merz, C.J. (1998). UCI Repository of machine learning databases [http://www.ics.uci.edu/~mlearn/MLRepository.html]. Irvine, CA: University of California, Department of Information and Computer Science.
gridGraphics	Paul Murrell and Zhijian Wen (2020). gridGraphics: Redraw Base Graphics Using 'grid' Graphics. R package version 0.5-1. https://CRAN.R-project.org/package=gridGraphics

cowplot	Claus O. Wilke (2020). cowplot: Streamlined Plot Theme and Plot Annotations for 'ggplot2'. R package version 1.1.1. https://CRAN.R-project.org/package=cowplot
ggpubr	Alboukadel Kassambara (2020). ggpubr: 'ggplot2' Based Publication Ready Plots. R package version 0.4.0. https://CRAN.R-project.org/package=ggpubr
gtable	Hadley Wickham and Thomas Lin Pedersen (2019). gtable: Arrange 'Grobs' in Tables. R package version 0.3.0. https://CRAN.R-project.org/package=gtable
data.table	Matt Dowle and Arun Srinivasan (2021). data.table: Extension of `data.frame`. R package version 1.14.0. https://CRAN.R-project.org/package=data.table
stringr	Hadley Wickham (2019). stringr: Simple, Consistent Wrappers for Common String Operations. R package version 1.4.0. https://CRAN.R-project.org/package=stringr
randomForest	A. Liaw and M. Wiener (2002). Classification and Regression by randomForest. R News 2(3), 18--22.
mlbench	Newman, D.J. & Hettich, S. & Blake, C.L. & Merz, C.J. (1998). UCI Repository of machine learning databases [http://www.ics.uci.edu/~mlearn/MLRepository.html]. Irvine, CA: University of California, Department of Information and Computer Science.
caret	Max Kuhn (2020). caret: Classification and Regression Training. R package version 6.0-86. https://CRAN.R-project.org/package=caret
rlist	Kun Ren (2016). rlist: A Toolbox for Non-Tabular Data Manipulation. R package version 0.4.6.1. https://CRAN.R-project.org/package=rlist
FactoMineR	Sebastien Le, Julie Josse, Francois Husson (2008). FactoMineR: An R Package for Multivariate Analysis. Journal of Statistical Software, 25(1), 1-18. 10.18637/jss.v025.i01
grid	R Core Team (2021). R: A language and environment for statistical computing. R Foundation for Statistical Computing, Vienna, Austria. URL https://www.R-project.org/.
ggforce	Thomas Lin Pedersen (2021). ggforce: Accelerating 'ggplot2'. R package version 0.3.3. https://CRAN.R-project.org/package=ggforce
randomcoloR	Ron Ammar (2019). randomcoloR: Generate Attractive Random Colors. R package version 1.1.0.1. https://CRAN.R-project.org/package=randomcoloR
scatterplot3d	Ligges, U. and Mächler, M. (2003). Scatterplot3d - an R Package for Visualizing Multivariate Data. Journal of Statistical Software 8(11), 1-20.
gridBase	Paul Murrell (2014). gridBase: Integration of base and grid graphics. R package version 0.4-7. https://CRAN.R-project.org/package=gridBase
nnet	Venables, W. N. & Ripley, B. D. (2002) Modern Applied Statistics with S. Fourth Edition. Springer, New York. ISBN 0-387-95457-0
reshape2	Hadley Wickham (2007). Reshaping Data with the reshape Package. Journal of Statistical Software, 21(12), 1-20. URL http://www.jstatsoft.org/v21/i12/

scatterplot3d	Ligges, U. and Mächler, M. (2003). Scatterplot3d - an R Package for Visualizing Multivariate Data. Journal of Statistical Software 8(11), 1-20
Rmisc	Ryan M. Hope (2013). Rmisc: Rmisc: Ryan Miscellaneous. R package version 1.5. https://CRAN.R-project.org/package=Rmisc
GGally	Barret Schloerke, Di Cook, Joseph Larmarange, Francois Briatte, Moritz Marbach, Edwin Thoen, Amos Elberg and Jason Crowley (2021). GGally: Extension to 'ggplot2'. R package version 2.1.1. https://CRAN.R-project.org/package=GGally
parcoords	Mike Bostock, Kai Chang, Xing Yun, Kenton Russell and Anobel Odisho (2019). parcoords: 'Htmlwidget' for 'd3.js' Parallel Coordinates Chart. R package version 1.0.0. https://CRAN.R-project.org/package=parcoords
viridis	Simon Garnier, Noam Ross, Robert Rudis, Antônio P. Camargo, Marco Sciaini, and Cédric Scherer (2021). Rvision - Colorblind-Friendly Color Maps for R. R package version 0.6.1.
SnowballC	Milan Bouchet-Valat (2020). SnowballC: Snowball Stemmers Based on the C 'libstemmer' UTF-8 Library. R package version 0.7.0. https://CRAN.R-project.org/package=SnowballC
lsa	Fridolin Wild (2020). lsa: Latent Semantic Analysis. R package version 0.73.2. https://CRAN.R-project.org/package=lsa
hrbrthemes	Bob Rudis (2020). hrbrthemes: Additional Themes, Theme Components and Utilities for 'ggplot2'. R package version 0.8.0. https://CRAN.R-project.org/package=hrbrthemes
